# The selective sponging of miRNAs by OIP5-AS1 regulates metabolic reprogramming of pyruvate in adenoma-carcinoma transition of human colorectal cancer

**DOI:** 10.1186/s12885-024-12367-7

**Published:** 2024-05-21

**Authors:** Jing-Yu Wang, Xiao-Ping Zhang, Hong-Kun Zhou, Hong-Xin Cai, Jin-Biao Xu, Bao-Gang Xie, Jean-Paul Thiery, Wu Zhou

**Affiliations:** 1 School of Medicine, Jiaxing University, No 118, Road Jiahang Avenue, Jiaxing, Zhejiang 314001 China; 2 Affiliated Hospital of Jiaxing University (The First Hospital of Jiaxing), Jiaxing, 314001 China; 3 Guangzhou laboratory, Guangzhou, 510700 China; 4 Institute of Molecular and Cell Biology, A-STAR, Singapore, 138673 Singapore

**Keywords:** RNA interactomes, Colorectal carcinoma, Metabolic reprogramming, Adenoma-carcinoma transition

## Abstract

RNA interactomes and their diversified functionalities have recently benefited from critical methodological advances leading to a paradigm shift from a conventional conception on the regulatory roles of RNA in pathogenesis. However, the dynamic RNA interactomes in adenoma-carcinoma sequence of human CRC remain unexplored. The coexistence of adenoma, cancer, and normal tissues in colorectal cancer (CRC) patients provides an appropriate model to address this issue. Here, we adopted an RNA in situ conformation sequencing technology for mapping RNA-RNA interactions in CRC patients. We observed large-scale paired RNA counts and identified some unique RNA complexes including multiple partners RNAs, single partner RNAs, non-overlapping single partner RNAs. We focused on the antisense RNA OIP5-AS1 and found that OIP5-AS1 could sponge different miRNA to regulate the production of metabolites including pyruvate, alanine and lactic acid. Our findings provide novel perspectives in CRC pathogenesis and suggest metabolic reprogramming of pyruvate for the early diagnosis and treatment of CRC.

**Supplementary Information:**

The online version contains supplementary material available at 10.1186/s12885-024-12367-7.

## Introduction

RNA is considered as the earliest molecular form at the origin of life. RNA not only carries genetic information such as DNA, but also has catalytic functions normally found in enzymes. The central position of RNA in cellular processes and gene regulation reflects its biochemical characteristics and early expression in the evolutionary process [1]. In cells, most RNAs form a secondary structure through intramolecular base pairing, and further fold to form a complex tertiary structure mediated by RNA binding proteins. Then, highly structured RNA interacts with other RNA molecules to play a biological regulatory role. Previous studies on RNA interactions have shown that the ability of RNA to interact with its own (intramolecular) and other (intermolecular) base pairs is critical to its function in the body [2]. Recognition of paired RNA-RNA interactions (RRI) is key to understanding how RNA functions dynamically change in cancer [3].

Colorectal cancer (CRC) is one of the most common cancers in the world and the third leading cause of cancer related deaths [4]. Although there is a strong genetic component, most colorectal cancer cases are sporadic and develop slowly through an adenoma-carcinoma sequence within a few years [5]. The transition from adenoma to carcinoma has been proposed as a critical step in colorectal carcinogenesis. The presence of adenoma, cancer and normal mucosa samples in CRC patients provides an appropriate model for the study of carcinogenesis and molecular events in the tumorigenic process. In recent years, the overall effects of the environment, lifestyle factors [6], and inherited and acquired genetic/epigenetic alterations [7, 8] on CRC, and the interactions between them have been clarified. However, how RNAs fold and interact with other RNAs during adenoma-carcinoma sequence have not been systematically investigated.

RIC-seq, the RNA in situ conformation sequencing technology, was previously designed as a tool to capture the global profiling of RNA–RNA interactions [9]. Recently, RIC-seq was applied to analyze SARS-CoV-2 RNA in situ structures and interactions in infected cells and patient lung samples [10], and extended as capture RIC-Seq (CRIC-seq) to enrich specific RNA binding protein (RBP)-associated in situ proximal RNA-RNA fragments for deep sequencing [11]. Here, we adopted RIC-seq technology mapping RNA interactomes in adenoma-carcinoma sequence of human CRC. We aimed to clarify the comprehensive profiling of RRI in colorectal adenoma, CRC, and adjacent normal tissues and identify potential RRI key events along the colorectal adenoma to CRC transition. We focused on one antisense RNA OIP5-AS1 for its selective sponging different miRNA regulating the metabolism of pyruvate. Our findings provide novel perspectives in CRC pathogenesis and suggest possible metabolic reprogramming of pyruvate for the early diagnosis and treatment of CRC.

## Results

### Global view of RNA interactomes in colorectal carcinoma patients

**Fig. 1:**
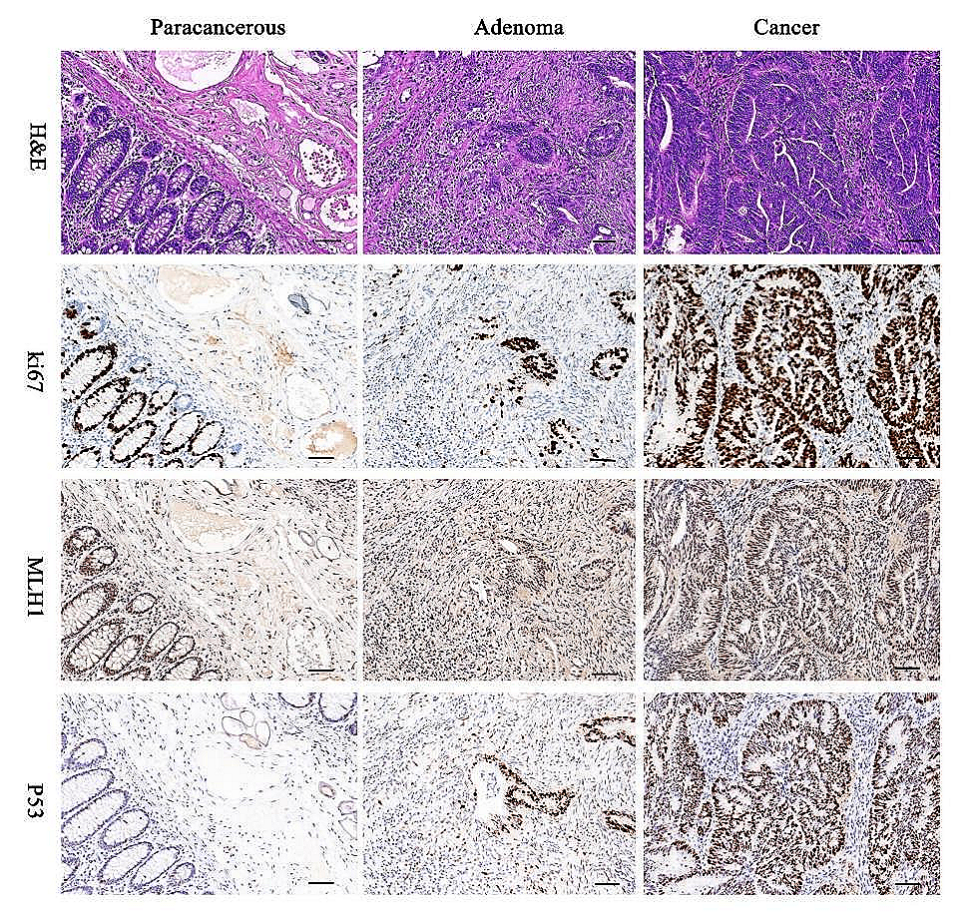
The adenoma-carcinoma sequence in colorectal cancer patients. H&E and immunohistochemistry for ki67, MLH1 and P53 were performed on the paracancerous, adenomas and cancer tissues of CRC patients. Representative images were displayed. Magnification, 10×. Scale bar, 40 μm

To study the dynamics of RNA interactomes, RIC-seq [9] (Supplementary Fig. 1) was performed on several biological replicates of colorectal carcinoma patient specimens, each including adenoma, malignant tissues, and normal tissues distal to cancer (Fig. 1 and Supplementary data file 1). Based on almost one billion sequencing reads (Supplementary Table 1), we identified > 9,000 intermolecular and > 3,000 intramolecular interactions and most uniquely mapped reads across different tissues (Supplementary Table 2). We observed a high correlation between biological replicates (*R* = 0.7–0.9), confirming that RIC-seq data are reproducible (Supplementary Fig. 2).

**Fig. 2:**
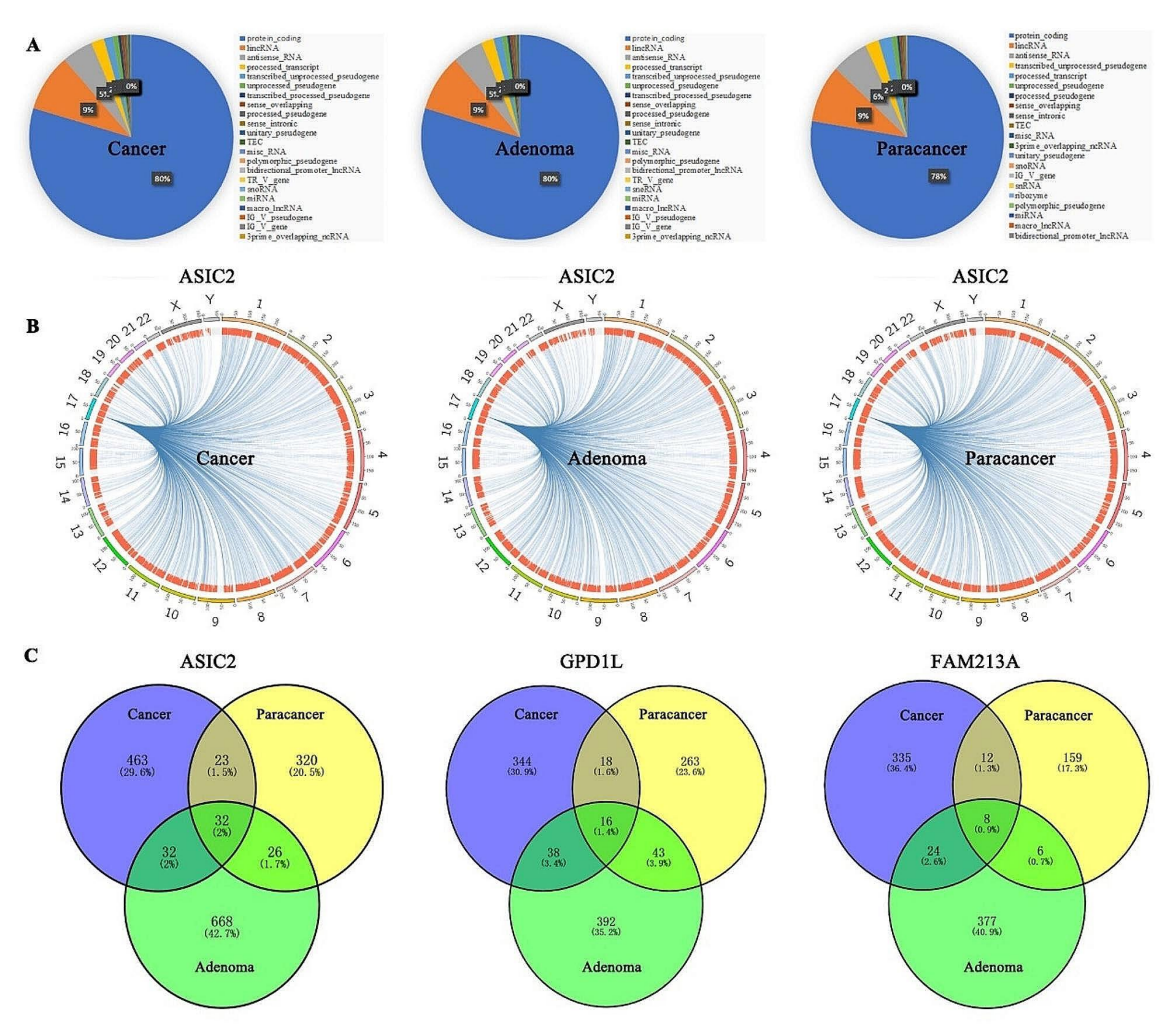
Global view and difference of RNA-RNA interactions during Adenoma-carcinoma Sequence. (**A**). The RNA types involved into RNA-RNA interactions in the samples of cancer, adenoma and paracancer. (**B**). Circos plot showing the ASIC2-interacting RNAs. The outer circles show different chromosomes, and the inner circles show unique contacts. (**C**). Venn diagram showing the RNA interactors of multiple partners RNA ASIC2, GPD1L and FAM213A among the samples of cancer, adenoma and paracancer

Next, we defined Monte Carlo simulation approach [12] for reliable intermolecular interactions. The observed paired counts were compared with analog counts to determine significant proximal interactions (Supplementary Fig. 3A). Overall, 16,168, 17,592, 18,151 RNA interactions were captured in the cancerous, adenoma and paracancerous tissues, respectively (Supplementary data file 2). After removing the data of interaction between two identical RNAs, the remaining types of interaction were remarkably diverse, including protein coding RNA to protein coding RNA, protein coding RNA to lincRNA, lincRNA to lincRNA, among others (Supplementary Fig. 3B). A rich resource of RNA was involved into the interactions, varying from unprocessed pseudogene, unitary pseudogene, transcribed unprocessed pseudogene, transcribed processed pseudogene, processed pseudogene, polymorphic pseudogene, to protein coding, processed transcript, and snoRNA, lincRNA, antisense RNA, bidirectional promoter lncRNA (Fig. 2A and supplementary Table 3).

### Difference of RNA-RNA interactions during adenoma-carcinoma sequence

To further explore the characteristics of RNA interaction, we listed in detail all RNA pairs that interact in paracancerous, adenoma and cancerous tissues (Supplementary data file 3) and found that one group of RNA molecules was extraordinarily active and could interact with multiple RNAs in each kind of tissue sample (Fig. 2B and supplementary Fig. 4). We nominated this class of RNAs as multiple partner RNA (MP-RNA) and compared their interacting RNA couple numbers in different tissues (supplementary Table 4). Moreover, the targeting RNAs of MP-RNA were thoroughly examined among paracancerous, adenoma and cancerous tissues. Our results showed that, whether in the samples of cancer, adenoma or paracancer, the top three MP-RNA counted by partner numbers were coding for ASIC2, GPD1L and FAM213A, indicating that these three RNAs were always active in both normal and abnormal colon tissues (Supplementary Tables 4 and Supplementary data file 4). These three MP-RNA have hundreds of partners in each sample, which may be located on the same or different chromosomes (supplementary Fig. 5). However, except for a small proportion of the overlap in the two samples (cancer V.S paracancer, cancer V.S adenoma or adenoma V.S paracancer), the common RNA partners of these three MP-RNAs (ASIC2, GPD1L and FAM213A) in all three samples (cancer, paracancer and adenoma) were exceedingly rare (from 0.9 to 2%), and the vast majority of their RNA partners differed from each other (Fig. 2C and Supplementary data file 4).

Noteworthy, the numbers of RNA interactors of some MP-RNAs (WDR62, TGFBR3, RN7SL5P, RMRP, MIR663AHG, AJ009632.2, CDKL1, OPRD1, PLOD1, AL117692.1, ATP13A2, EPB41) in cancer tissue were significantly increased compared with that in paracancer and adenoma. Some MP-RNAs interacted with much more partners in adenoma and cancer tissues than in paracancer tissues, such as SLC16A14, POLA2, PCDH11X, TASP1, TSPY20P. Some MP-RNAs (ARID4B, CPT1A, PHKB) had increased number of partners in adenoma samples than in the paracancer samples but decreased in cancer samples (Supplementary data file 4). These data revealed that the activity of MP-RNAs in different samples was variable, and even the same MP-RNA had its own preference to select partners across different tissues.

In addition to MP-RNAs, another special type of RNA was identified and named as single partner (SP) RNA, which had only one interactor in each kind of tissue sample. More specifically, the interacting elements of one group of SP-RNAs did not overlap in different tissues (Supplementary data file 5). Among these non-overlapping single partner (NSP) RNAs, we were attracted by an unusual antisense RNA whose symbol was OIP5-AS1 and was identified to bind miRNA-873, miRNA-335 and miRNA-323a in samples of paracancer, adenoma and cancer, respectively (Supplementary data file 5).

### The selective binding of miRNAs by OIP5-AS1

**Fig. 3:**
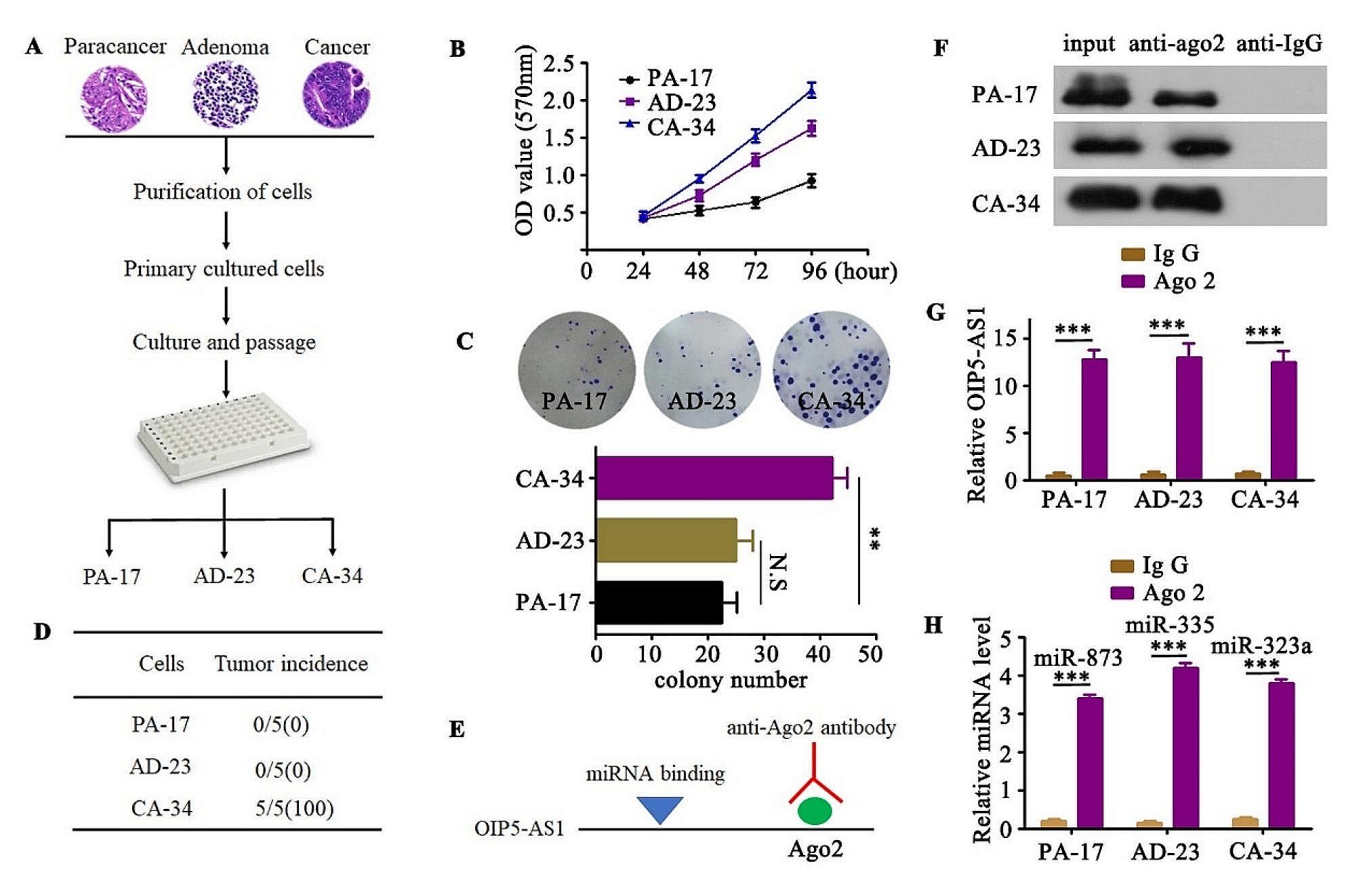
The selective binding of miRNAs by OIP5-AS1. (**A**). The scheme for establishing PA-17, AD-23 and CA-34 cells. (**B** and **C).** Proliferation and invasive capacities of PA-17, AD-23 and CA-34 cells were assessed by MTT (B) and in vitro invasion assay (**C**), respectively. Data were quantified and presented as the mean ± SD of triplicate experiments. N.S, no significance; **P < 0.001. (**D**). PA-17, AD-23 or CA-34 cells were subcutaneously transplanted into recipient nude mice. Tumor incidence, described with tumors/transplanted mice (ratio), was measured at 8 weeks after transplantation. (**E**). Schematic outlining the Ago2 RIP strategy to validate endogenous miRNA with OIP5-AS1 binding. (**F**). The Ago2 antibody precipitated the Ago2 protein from cell lysates from PA-17, AD-23 or CA-34 cells, while the control non-immune IgG did not. The input lane indicates lysate samples used as a positive control for Ago2. The original gel data were showed in supplementary Fig. 9A. (**G**). qRT-PCR assessment of OIP5-AS1 in PA-17, AD-23 or CA-34 cells that were pulled down by Ago2 or negative control IgG and normalized to U6 snRNA. (**H**). qRT-PCR detection of miR-873, miR-335 or miR-323a endogenously associated with OIP5-AS1 in PA-17, AD-23 or CA-34 cells that were pulled down by AGO2 or negative control IgG and normalized to U6 snRNA. Data are means + SD from three independent experiments. ***P < 0.001

To validate the interactions between OIP5-AS1 and their miRNA patners, we established a series of cell lines by limiting dilution from tissues of paracancer, adenoma or cancer (Fig. 3A). Firstly, the malignant potential of tissue-derived PA-17 (paracancer), AD-23(adenoma) and CA-34 (cancer) cells were examined. Compared with PA-17 cells, AD-23 cells had no stronger invasion ability in vitro, but the proliferation ability of cells was enhanced (Fig. 3B and C). The PA-17, AD-23 and CA-34 cells were further transplanted subcutaneously into recipient nude mice. The results showed that only CA-34 cells transplanted mice formed palpable tumors post-transplantation (Fig. 3D). These data suggest that cancer tissue-derived CA-34 cells had the ability of malignant transformation, whereas AD-23 and PA-17 cells had not yet acquired a malignant phenotype.

Then, RNA immunoprecipitation (RIP) assays using antibodies against mouse Ago2 (Fig. 3E), which is a core component of the RNA-induced silencing complex (RISC) [13], were further employed to validate the interactions. Ago2 antibody precipitated Ago2 protein-RNA complexes from PA-17, AD-23 and CA-34 cell lysates (Fig. 3F), and we found that endogenous OIP5-AS1 was preferentially enriched in Ago2 RIPs compared to control immunoglobulin G (IgG) antibody RIPs (Fig. 3G). Moreover, Ago2 RIP samples from PA-17, AD-23 and CA-34 cells were significantly enriched for endogenous miRNA-873, miRNA-335 and miRNA-323a, respectively (Fig. 3H), demonstrating the characteristics of OIP5-AS1 selectively binding to different miRNAs.

### The regulation of metabolites by OIP5-AS1 and their miRNA partners

**Fig. 4:**
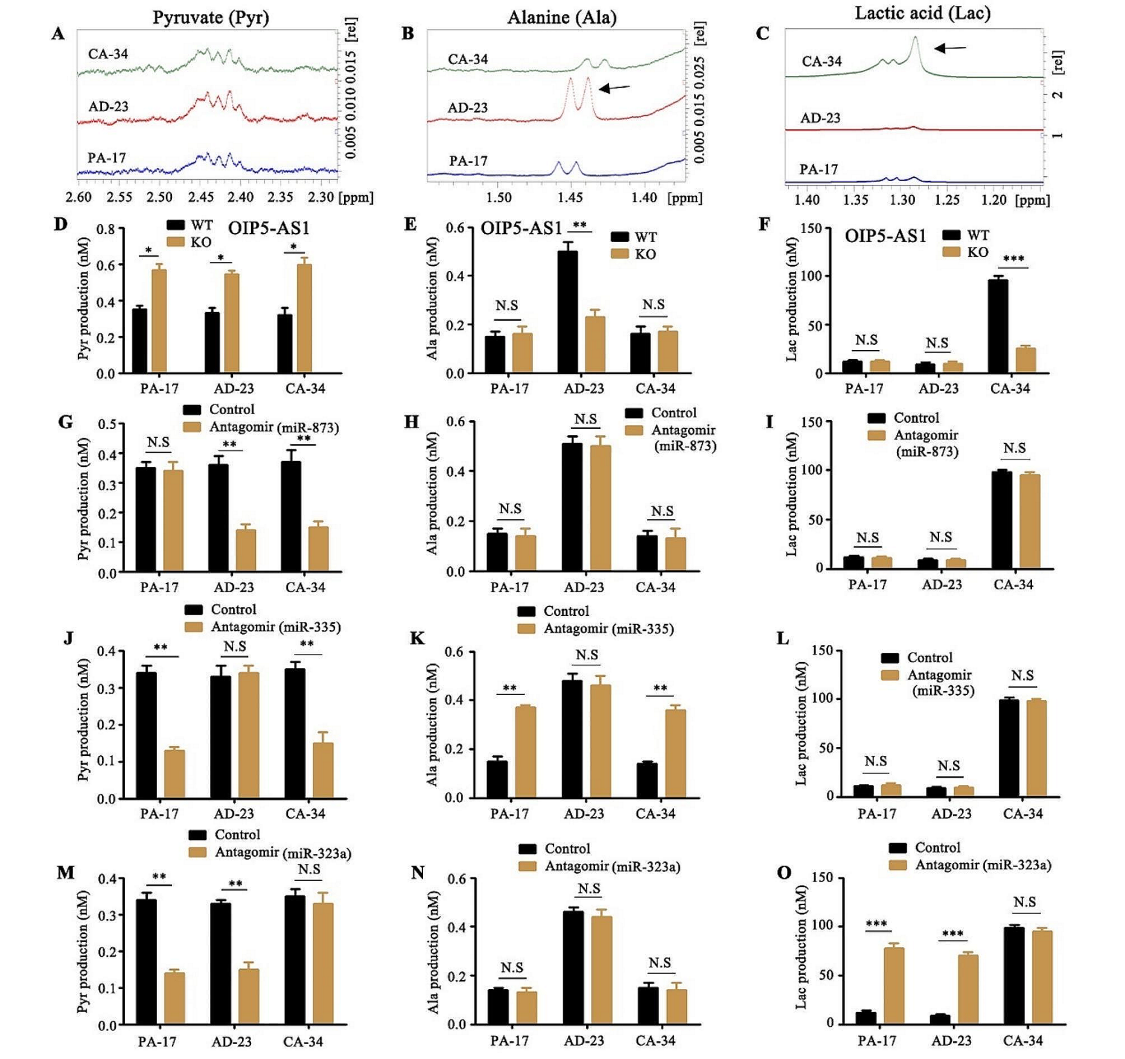
The regulation of metabolites by OIP5-AS1 and their miRNA partners. (**A, B** and **C**). The signal in relative chemical shifts between 2.30–2.60 ppm (A, pyruvate), 1.40–1.50 ppm (B, alanine) and 1.20-1.40ppm (C, lactic acid) were selected to export the original data. The arrows show the sites of metabolite with density variation. (**D, E** and **F**). The production of pyruvate (D), alanine (E) or lactic acid (F) in OIP5-AS1 knock out (KO) or wild type (WT) PA-17, AD-23 or CA-34 cells was detected by Colorimetric Assay kits. (**G - O**). The antagomirs or controls of miR-873 (G, H and I), miR-335 (J, K and L) and miR-323a (**M, N** and **O**) were individually transfected into PA-17, AD-23 or CA-34 cells. The production of pyruvate (**G, J** and **M**), alanine (**H, K** and **N**) or lactic acid (**I, L** and **O**) were measured by Colorimetric Assay kits. Data are means + SD from three independent experiments. ***P < 0.001, **P < 0.01, *P < 0.05, N.S, no significance

After confirming the interactions between OIP5-AS1 and their non-overlapping miRNA partners, we ask to understand what functions are affected by these selective bindings. The targets of miRNA-873, miRNA-335 and miRNA-323a in RIC-seq (Supplementary Table 5) were analyzed by Gene Ontology (GO) enrichment, showing that most of enriched functions were associated with metabolism (Supplementary Fig. 6A). Thus, we investigated the metabolic characteristics of PA-17, AD-23 and CA-34 cells by ^1^HNMR analysis. The results revealed that the density of metabolites was varied among different samples detecting by relative chemical shift between 0 and 10 ppm (Supplementary Fig. 6B). In particular, the ^1^HNMR spectrum showed that the metabolite alanine was promoted in AD-23 cells, while lactic acid had a high yield in CA-34 cells (Fig. 4A, B and C). Additionally, the production of alanine was highly increased in adenoma tissues instead of cancerous or paracancerous tissues and tissue sample of cancer produced much more lactic acid than that of adenoma or paracancer (Supplementary Fig. 7A-7D).

To test whether the outputs of the metabolite alanine, pyruvate and lactic acid were determined by OIP5-AS1, we knocked out OIP5-AS1 in PA-17, AD-23 and CA-34 cells separately. Our results showed that the metabolic level of alanine in AD-23 cells or that of lactic acid in CA-34 cells was remarkably reduced with the deletion of OIP5-AS1. However, the metabolite pyruvate was induced in OIP5-AS1 knocking out PA-17, AD-23 and CA-34 cells, compared with control cells (Fig. 4D, E and F).

Besides, the role of OIP5-AS1 interacting miRNA partners in the regulation of the metabolite alanine, pyruvate and lactic acid were evaluated. The RNA levels of miR-873, miR-335 and miR-323a did not exhibit significant difference among PA-17, AD-23 and CA-34 cells (Supplementary Fig. 7E). The antagomirs or controls of miR-873, miR-335 and miR-323a were individually transfected into these three cell lines. The results reflected a modest complexity that miR-873 antagomir inhibited the pyruvate production in AD-23 and CA-34 cells but had no effect in PA-17 cells. In addition, the production of alanine and lactic acid in PA-17, AD-23 and CA-34 cells was not changed due to the presence of miR-873 antagomir (Fig. 4G, H and I). On the other hand, miR-335 antagomir promoted the metabolism of alanine in PA-17 and CA-34 cells and reduced the level of pyruvate in them, while miR-323a antagomir significantly increased the production of lactic acid and the consumption of pyruvate in PA-17 and AD-23 cells (Fig. 4J and O). Together, we inferred that OIP5-AS1 and OIP5-AS1 coupled non-overlapping miR-873, miR-335 and miR-323a were involved into the regulation of metabolism of pyruvate, alanine and lactic acid.

### The regulation of miRNA target genes by the selective sponging of OIP5-AS1

Next, we investigated how OIP5-AS1 and their miRNA partners regulate the metabolism of pyruvate, alanine and lactic acid. Since the metabolites pyruvate, alanine and lactic acid are all associated with pyruvate metabolism and OIP5-AS1 is a conserved gene that produces a long non-coding RNA and can bind to and negatively regulate the activity of multiple cellular RNAs and microRNAs [14], we posited that the OIP5-AS1 might selectively sponge different non-overlapping miRNA partners and regulated pyruvate metabolism in different stages of colorectal cancer initiation and progression.

**Fig. 5:**
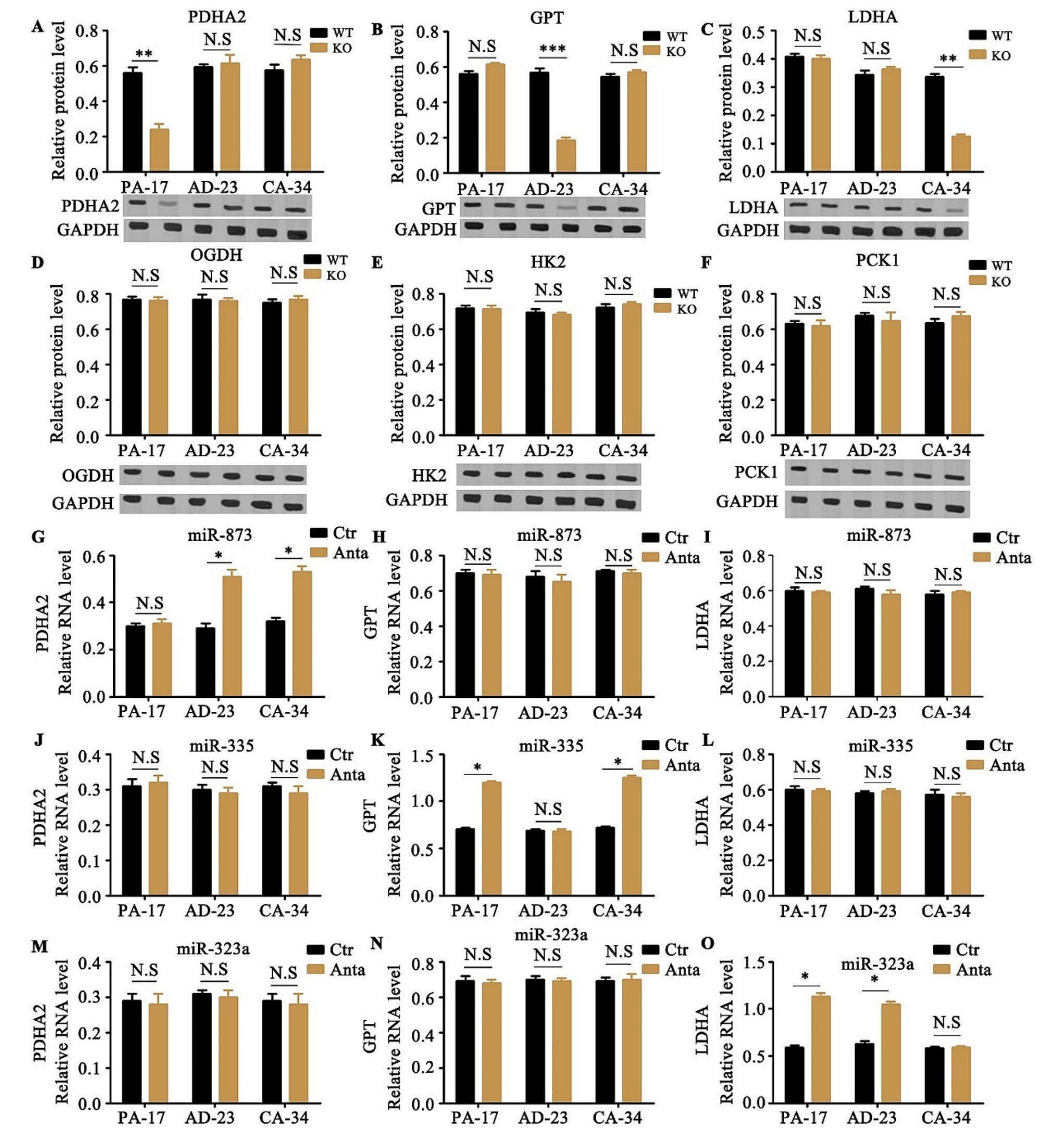
The regulation of miRNA target genes. (**A - F**). The protein expression of PDHA2 (**A**), GPT (**B**), LDHA (**C**), OGDH (**D**), HK2 (**E**) and PCK1 (**F**) were analyzed in OIP5-AS1 knocking out (KO) PA-17, AD-23 and CA-34 cells or control wild type (WT) cells by western blotting. The relative protein levels were quantified and normalized to GAPDH. The original gel data were showed in supplementary Fig. 8. (**G - O**). The antagomirs (Anta) or controls (Ctr) of miR-873 (**G**, **H** and **I**), miR-335 (**J**, **K** and **L**) and miR-323a (**M**, **N** and **O**) were individually transfected into PA-17, AD-23 or CA-34 cells. The RNA expression of PDHA2 (**G**, **J** and **M**), GPT (**H**, **K** and **N**) and LDHA (**I**, **L** and **O**) were measured by qRT-PCR. The relative RNA levels were quantified and normalized to U6 snRNA. Data are means + SD from three independent experiments. ***P < 0.001, **P < 0.01, *P < 0.05, N.S, no significance

**Fig. 6:**
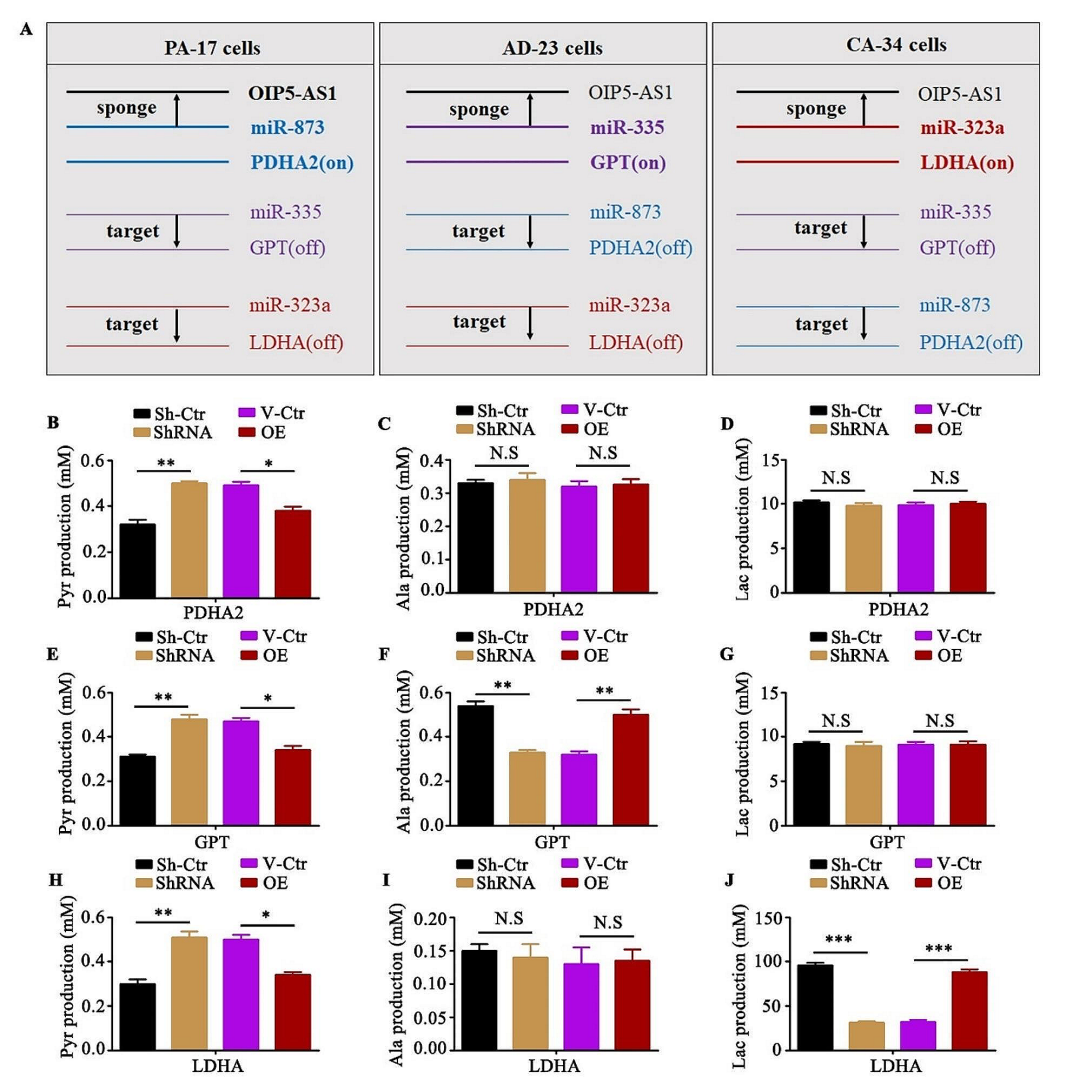
The selective sponging of miRNAs by OIP5-AS1. (**A**). The scheme for selective sponging of miR-873, miR-335 and miR-323a by OIP5-AS1. (**B, C** and **D**). The PDHA2 shRNA (ShRNA) or shRNA control (Sh-Ctr) plasmids were transfected into PA-17 cells and selected by puromycin. The stable clone cells with PDHA2 shRNA were transiently transfected with PDHA2 cDNA (OE) or vector control (V-Ctr) plasmids. The production of pyruvate (**B**), alanine (**C**) or lactic acid (**D**) were measured by Colorimetric Assay kits. (**E, F** and **G**). The GPT shRNA (ShRNA) or shRNA control (Sh-Ctr) plasmids were transfected into AD-23 cells and selected by puromycin. The stable clone cells with GPT shRNA were transiently transfected with GPT cDNA (OE) or vector control (V-Ctr) plasmids. The production of pyruvate (**E**), alanine (**F**) or lactic acid (**G**) were measured by Colorimetric Assay kits. (**H, I** and **J**). The LDHA shRNA (ShRNA) or shRNA control (Sh-Ctr) plasmids were transfected into CA-34 cells and selected by puromycin. The stable clone cells with LDHA shRNA were transiently transfected with LDHA cDNA (OE) or vector control (V-Ctr) plasmids. The production of pyruvate (**H**), alanine (**I**) or lactic acid (**J**) were measured by Colorimetric Assay kits. Data are means + SD from three independent experiments. ***P < 0.001, **P < 0.01, *P < 0.05, N.S, no significance

To test this hypothesis, several pyruvate metabolism related genes among supplementary Table 5 were further investigated. We deleted the RNA of OIP5-AS1 and found that the protein levels of PDHA2, GPT or LDHA were significantly decreased in PA-17, AD-23 or CA-34 cells, respectively, while the protein expression of OGDH, HK2 or PCK1 was not affected by the deletion of OIP5-AS1 in PA-17, AD-23 and CA-34 cells (Fig. 5A and F). PDHA2 belongs to the component of the human pyruvate dehydrogenase complex and catalyzes the overall conversion of pyruvate to acetyl-CoA and CO2, and thereby links the glycolytic pathway to the tricarboxylic cycle [15]. GPT, also known as GPT1 or ALT1, encodes cytosolic alanine aminotransaminase and catalyzes the reversible transamination between alanine and 2-oxoglutarate to generate pyruvate and glutamate and, therefore, plays a key role in the intermediary metabolism of glucose and amino acids [16]. LDHA is one of five isoforms of the lactate dehydrogenase family. It catalyzes the conversion of pyruvate to lactate under anaerobic conditions and is key in the altered glycolytic metabolism that is a feature of cancer cells [17]. The miRNA-873 antagomirs boosted the expression of PDHA2 in AD-23 and CA-34 cells (Fig. 5G, H and I), the inhibition of miRNA-335 increased GPT in PA-17 and CA-34 cells (Fig. 5J, K and L), and miRNA-323a antagomirs induced LDHA level in PA-17 and AD-23 cells (Fig. 5M, N and O), from which we inferred that miR-873 targeted PDHA2 and OIP5-AS1 sponged miR-873 in PA-17 cells, miR-335 targeted GPT and OIP5-AS1 sponged miR-335 in AD-23 cells, and miR-323a targeted LDHA and OIP5-AS1 sponged miR-323a in CA-34 cells (Fig. 6A).

Furthermore, we used shRNA plasmids to stably knock-down PDHA2 in PA-17 cells, GPT in AD-23 cells or LDHA in CA-34 cells. To some extent, PDHA2, GPT or LDHA shRNA significantly promoted the production of pyruvate, compared with shRNA control (Fig. 6B and J). However, GPT shRNA transfecting AD-23 cells produced less alanine (Fig. 6E, F and G) and the inhibition of LDHA in CA-34 cells gained fewer lactic acid (Fig. 6H, I and J). Most importantly, the decrease in alanine production mediated by GPT shRNA and the reduction in lactic acid caused by LDHA shRNA can be rescued after being transfected with exogenous GPT or LDHA cDNA (Fig. 6B and J). Together, we demonstrated that the selective sponging of miR-873, miR-335 or miR-323a in PA-17, AD-23 or CA-34 cells by OIP5-AS1 separately regulated the metabolism of pyruvate by PDHA2, GPT or LDHA.

## Discussion

**Fig. 7:**
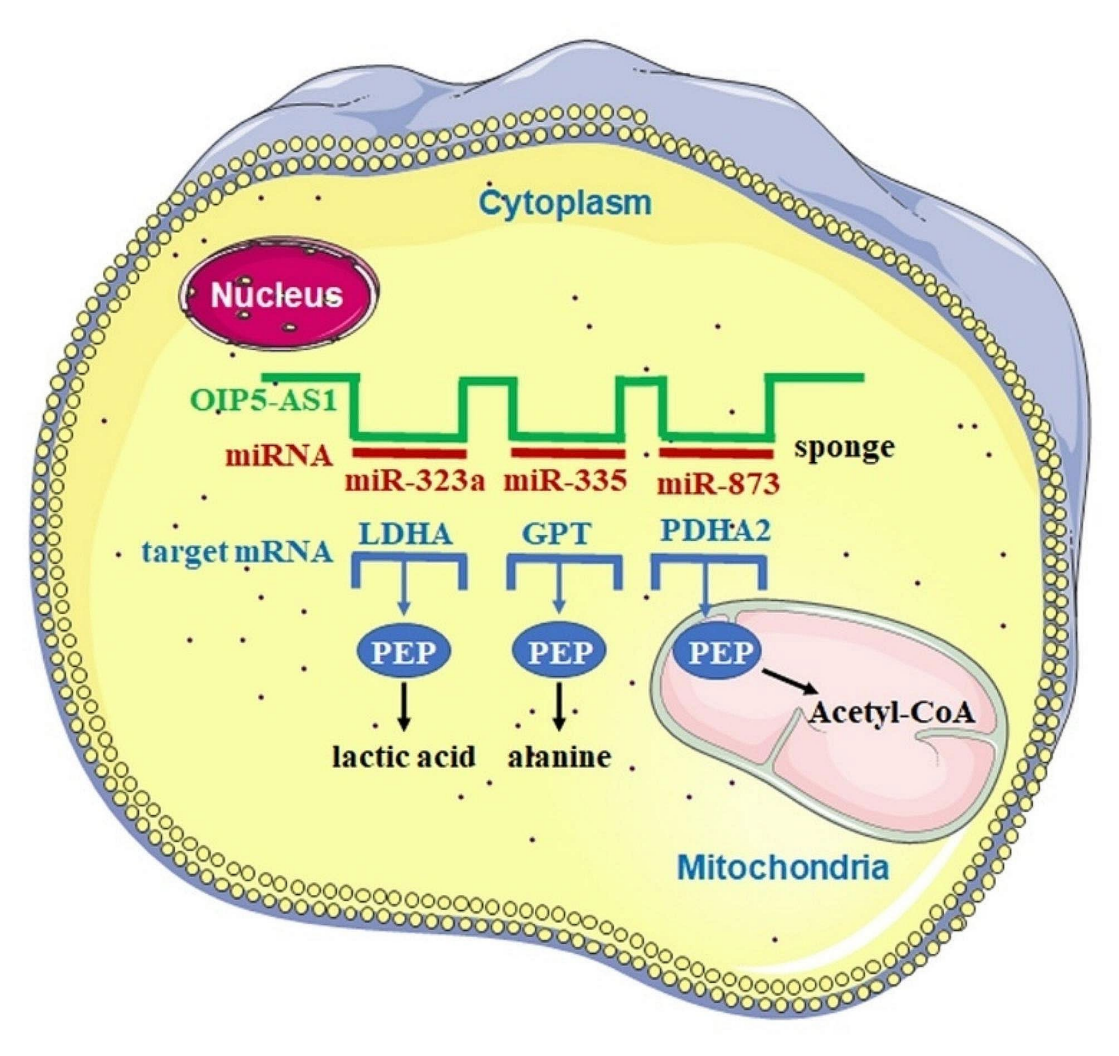
Proposed model for the regulatory mechanism mediated by OIP5-AS1

Metabolic reprogramming, a hallmark of cancer, is closely related to the initiation and progression of carcinoma. Adenoma, as a precursor state to carcinoma of colon cancer, provides a suitable model for studying the gradual evolution of metabolic reprogramming in tumorigenesis. Here we report that the metabolism of pyruvate was reprogrammed by the antisense RNA OIP5-AS1 in adenoma-carcinoma transition of CRC. Our results indicate that OIP5-AS1, under normal physiological conditions, sponge miRNA-873 in colonic cells, restoring to the maintenance of the expression of PDHA2 and allowing as the metabolism of pyruvate into acetyl-CoA. When the colonic mucosa transforms into an adenoma, the sponging by OIP5-AS1 shifts to miRNA-335 promoting the expression of GPT, which catalyzes pyruvate to alanine by transamination. After further transformation into cancer, OIP5-AS1 sponges miR-323a, leads to an increase in LDHA and thus reprogram the metabolism of pyruvate into lactic acid (Fig. 7).

The conversion of pyruvate to alanine in adenoma prior to lactic acid in cancer indicates the metabolic changes before complete transformation of cancer. As Fearon and Vogelstein (5) put forward in the adenoma‑carcinoma sequence model, colorectal cancer develops from adenoma through a series of genetic events, including gene mutations of oncogenes and loss of tumor suppressor genes [5]. The changes in cellular metabolism may precede the acquisition of driving mutations, ultimately leading to colon cell transformation. The morphologically normal colon tissue of mice with genetic susceptibility to CRC showed an increase in lactate [18] and changes in transcriptome were related to a variety of metabolic pathways, including glucose metabolism and insulin signal transduction [19, 20]. In rectal biopsy of normal tissues obtained from patients with one or more precancerous colonic lesions, the expression of HIF1A, GLUT1 and PKM2 was significantly increased, indicating that Glycolysis was upregulated [21]. The authors also found that the mitochondrial gene expression that promotes mitochondrial fission and fusion, uncoupled proton flow, ATP synthase activity, and mitochondrial copy number increased, indicating that OXPHOS changed [21].

Recent technological developments have made it possible to transform the field of RNA biology from studying one RNA at a time to mapping structures and interactions within the transcriptome [9, 22–25]. Sequencing based RRI studies have revealed the global prevalence and dynamics of RNA interaction networks and their impact on gene regulation. Our RIC-seq analysis on CRC patients identified some unexpected phenomena such as MP-RNAs. Why can one RNA interact with tens or hundreds of other RNAs in a cell? Why their RNA partners of such MP-RNAs are unstable in different tissues? How this kind of MP-RNAs act with their partners and what functions do they regulate? All these questions need further systematic research.

Another interesting phenomenon revealed by our RIC-seq analysis is that some family genes have homophilic interactions between one RNA and other RNAs from the same family member. For example, PCDHG, the protocadherin gamma gene cluster, includes 22 genes divided into 3 subfamilies, PCDHGA, PCDHGB and PCDHGC. Our data showed that RNA from 12 PCDHGA genes (A1-A12), 7 PCDHGB genes (B1-B7) and 2 PCDHGC genes (C3 and C4) could interact with each other in paracancer sample. However, in cancer tissue samples, only 5 PCDHGA genes (A1-A5) and 3 PCDHGB genes (B1-B3) showed homophilic RNA interactions. The partners of RNA from other 7 PCDHGA genes (A6-A12) and 4 PCDHGB genes (B4-B7) changed as unprocessed pseudogene AC034205.2 and protein coding gene OAZ3 (Supplementary data file 6). The switch of the manifestation of homophilic interactions in adenoma-carcinoma transition should connect to some mechanism for initiation or progression of human CRC, which will be systemically investigated in a future study.

Identifying paired RRIs is key to understanding how RNA folds and interacts with other RNAs within the cell. Tumor specific RRI can provide very important functional biomarkers, which can provide more relevant phenotypic features than currently available tumor specific biomarkers. Here, we investigated the role of RRIs in adenoma-carcinoma transition of human colorectal cancer. The functional assessment of other RRIs still needs to be explored in the future. The specific RRI spectrum in adenoma-carcinoma transition of CRC may help design new diagnostic and treatment strategies for CRC.

## Materials and methods

### Patient eligibility

This study included three colorectal cancer patients treated at The First Hospital of Jiaxing from August 2022 to December 2022 (Supplementary data file 1) and met the following requirements: (i) histologically diagnosed with colorectal cancer, (ii) not receiving treatment before diagnosis, and (iii) voluntarily signing a written informed consent form. The colorectal adenomas, cancerous tissues, and tissues distal to cancer collected from each specimen were applied for analysis of H&E, immunohistochemistry, RIC-seq and metabolites.

### Cell lines establishing

The tissues of adenoma, malignant tissues, and normal tissues distal to cancer from one CRC patient (#22-01707) who received surgical treatment at The First Hospital of Jiaxing were collected and individually dissected into smaller fragments. The tissue fragments were placed on a 10 cm culture plate and cultured in Dulbecco’s Modified Eagle’s Medium (DMEM) (Hyclone, SH30019.01, logan, UT) with 10% Fetal Bovine Serum (FBS). After the primary cells migrating out the tissue fragments, the fibroblasts are scraped off manually under the microscope. The remained epithelial cells were cultured and passaged for three generations. Then, by limiting dilution with 96 well plate, the isolated single cells were further amplified into single cell clones.

### RIC-seq

RIC-seq was performed as described in published protocol [9, 23]. Briefly, the indicated tissues were cross-linked with 1% formaldehyde for 20 min at room temperature (RT) and quenched by adding 1/20 volume of 2.5 M glycine. RNAs were then randomly cleaved by micrococcus nuclease and dephosphorylation at their 3′ end. After alkaline phosphatase treatment, the 3’ end of proximal interacting RNA fragments were labeled with biotinylated cytidine (bis) phosphate (pCp-biotin, Thermo Fisher, 20,160) using T4 RNA ligase (Thermo Fisher, EL0021) and subsequently ligated together under in situ and native conditions overnight. In the subsequent in vitro stage, the total RNA was extracted and fragmented, and the RNA containing C-biotin was enriched and then converted into paired-end libraries for deep sequencing on the Illumina NovaSeq 6000 System.

### H&E staining

The paracancerous, adenomas and cancer tissues of CRC patients were collected for H&E staining as previously described [26]. Briefly, 10-µm-thick sections were first incubated in Haematoxylin A solution for 3 min. After washing with water three times, the sections were rinsed in a concentrated HCl solution diluted with 70% ethanol for one minute and washed again with water three times. Then, the sections were incubated in 1% ammonia water for 1 min, washed for 3 times, stained with Eosin-Y solution for 8–10 s, dehydrated in a series of ethanol and xylene and mounted with neutral balsam. Olympus microscope (Olympus, DP72) was applied for images acquiring.

### Immunohistochemistry

The paracancerous, adenomas and cancer tissues of CRC patients were collected for immunohistochemistry (IHC) as previously described [27]. Briefly, the sample was fixed in 4% PFA at 4 °C for 1 h and washed 3 times with PBS. Then, the tissue was placed overnight in a 30% sucrose/PBS solution at 4 ° C. Afterwards, the samples were embedded in OCT compound (Sakura) and sliced. Seal the sections with PBSST (0.1% Triton X-100/2.5% normal donkey serum/PBS) at room temperature for 30 min, and then incubate them overnight with primary antibodies at 4 °C. Wash with PBS three times and incubate with the second antibody at room temperature for 30 min. Wash the slices three times with PBS and install them with media containing DAPI.

### shRNAs, miRNA antagomirs and plasmids

GPT shRNA, PDHA2 shRNA, LDHA shRNA and shRNA control were order from ThermoFisher Scientific Inc. The target sequences and double strand oligos were listed in Supplementary data file 7. The human miR-873 antagomir, miR-335 antagomir, miR-323a antagomir and control antagomir were purchased from Shanghai GenePharma Co., Ltd (Shanghai, China). The human cDNA open reading frame clone for PDHA2 (#HG20162-UT), LDHA(#HG16141-UT), GPT(#HG17947-UT) and pCMV3 vector control plasmids were purchased from Sino Biological, Inc.

For shRNA transfection, GPT shRNA, PDHA2 shRNA, LDHA shRNA or shRNA control plasmids were mixed with lipofectamine 3000 and transfected into indicated PA-17, AD-23 or CA-34 cells seeded into 24 well plates. 48 h later, the medium was replaced by DMEM with 10% FBS and 2.5 mg/ml puromycin for positive selection. The stable positive clones were sent for further verification by western blotting.

For miRNA antagomirs and plasmids transfection, the cDNA or control plasmids of GPT, PDHA2 and LDHA, antagomirs or controls of miR-873, miR-335 and miR-323a were individually mixed with lipofectamine 3000 and transiently transfected into indicated cells. The transfection efficiency was verified by qRT-PCR or western blotting.

### CRISPR/Cas9 mediated OIP5-AS1 knockout

OIP5-AS1 was knocked out in PA-17, AD-23 or CA-34 cells with the Smart-CRISPR™ gene editing system accomplished by Cyagen Biosciences Co., Ltd (Suzhou, China). Briefly, the sequence of OIP5-AS1 was obtained from the NCBI database, and the suitable sgRNA plasmids were designed and constructed. Using the CRISPR/Cas9 gene editing technology, Cas9 and sgRNA plasmids were transferred into PA-17, AD-23 or CA-34 cells by Lipofectamine® 3000 Transfection reagent (ThermoFisher, Langenselbold Germany; #L3000015). One day later, the transfected cells were selected with puromycin (concentration 5 ng/L). Thereafter, single-cell clones were growed in a 96-well plate and PCR and Sanger sequencing were performed to detect the knock-out (Supplementary data file 7).

### Ago2 RIP assay

To determine whether the OIP5-AS1, miR-873, miR-335 and miR323a are associated with the RISC, we performed RNA pull-down assay using Ago2 antibody to detect the RISC and OIP5-AS1 or miRNAs from the pellet using qRT-PCR. Briefly, rinse the indicated cells with cold PBS and fix with 1% formaldehyde for 10 min. After centrifugation, the precipitate was collected and resuspended in NP-40 pyrolysis buffer containing 1 mM phenylmethylsulfonyl fluoride (PMSF), 1 mM dithiothreitol, 1% protease inhibitor cocktail (Sigma-Aldrich), and RNase inhibitor (200 U/ml) (Life Technologies). After cell lysis, the supernatant is collected by high-speed centrifugation and stored at − 80 °C before use.

### Animal studies

Nude mice strains were bred by the Animal Center of Jiaxing University. One million PA-17, AD-23 or CA-34 cells were injected subcutaneously into six-week-old nude mice. Tumor incidence was measured at 8 weeks after transplantation.

### RNA extraction and qRT-PCR

Total RNA was isolated by TRIzol Reagent (Invitrogen) following the manufacturer’s instructions. Reverse transcription of RNA into cDNA using PrimeScript RT kit (Takara Biotechnology Co., Ltd.). Using cDNA as a template, SYBR Green Master mix (Roche), PCR forward primer (10 µM), Polymerase chain reaction reverse primer (10 µM) and sterile distilled water as raw materials, quantitative real time PCR amplification was performed under the following conditions: 95 ˚C pre denaturation for 10 min; 40 cycles of denaturation at 95˚C for 10 s, at 60˚C for 20 s. The relative fold changes of candidate genes were analyzed by using the 2 − DDCT method. U6 snRNA were applied as control.

### MTT assay

The indicated cells were inoculated at a density of 5000 cells per well in a 96 well plate, and add MTT solutions (Thiazolyl Blue Tetrazolium Bromide, Sigma, St. Louis, USA) to the sample after 24, 48, 72, or 96 h of cell growth. Dimethyl sulfoxide (DMSO, Sigma, St. Louis, USA) was added at 100 µL/well and plates were incubated in 37ºC incubator for 20 min. Measure the absorbance at a wavelength of 570 nm using a microplate reader (Bio-Rad, CA, USA).

### In vitro invasion assay

The invasive ability of PA-17, AD-23 or CA-34 cells was measured using 24-well Transwell plates (8-mm pore size; Corning), as previously described [27]. Briefly, the chamber inserts were coated with Matrigel (BD Biosciences) at 1:7 dilution and 5 × 10^4^ cells were collected in 200 ml DMEM and added to the upper chamber of the Transwell with a noncoated membrane.

### Western blotting

Cells were collected in lysis buffer containing Complete Protease Inhibitor Cocktail (Roche) and applicated to SDS-PAGE. Cellular proteins were transferred onto a nitrocellulose membrane (Millipore) and probed for anti-OGDH (#ab307369, Abcam; dilution 1:1000), anti-LDHA (#sc-133,123, Santa Cruz Biotechnology; dilution 1:1000), anti-GPT (#sc-374,501, Santa Cruz Biotechnology; dilution 1:1000), anti-HK2 (#ab273721, Abcam; dilution 1:2000), anti-PDHA2 (#TA379757, OriGene Technologies, Inc.; dilution 1:1000), anti-PCK1 (#ab133603, Abcam; dilution 1:2000) or anti-GAPDH (#9484, Abcam; dilution 1:5000) antibodies. After washing for 3 times with PBS, the membranes were incubated with secondary antibodies. The reaction was measured using an ECL chemiluminescence kit (Amersham Biosciences) with Eastman Kodak Co. hyper film. The gel blots were quantified by Multi Gauge software (Fujifilm Life Sciences) and presented as the mean ± SD, based on at least three independent experiments.

#### Metabolites measurement

Metabolites from indicated cells or tissues were measured by colorimetric assay kit for alanine (#E-BC-K130-M, Elabscience Technologies, Inc.), lactic acid (#E-BC-K044-M, Elabscience Technologies, Inc.) and pyruvate(#E-BC-K318-M, Elabscience Technologies, Inc.) following the instruction of the manufacturers. For tissues: take 0.02–0.2 g fresh tissue blocks were rinsed with PBS (0.01 M, pH 7.4) at 2–8 °C to remove blood then dry with filter paper and put it into the homogenization container. The homogenization medium was added according to the ratio of animal tissue weight (g): volume of homogenization medium (mL) = 1:9, and homogenization was performed at low temperature 4 °C, 10,000× G Centrifuge for 10 min. The supernatant was put on ice for test. For cells: the culture medium of cells was removed and washed with PBS (0.01 M, pH 7.4), and then collected by trypsin treatment. Cells were then resuspended in 2–5 mL PBS (0.01 M, pH 7.4).

#### ^1^H-NMR measurements

The tissue samples were thawed and crushed at room temperature and centrifuged in centrifuge tubes at 8000 rpm for 5 min. Take 150 µL supernatant, mixed by vigorous vortex with 800 µL of methanol, and then centrifuged at 14 000 g for 10 min. The supernatant was collected and concentrated by SpeedVac system at 35 °C and the residue was redissolved in 50.0 µL PBS, 450 µL distilled water and 50.0 µL TSP (0.5 mg/ml). The supernatant was centrifugated with 12 000 rpm for 10 min and transferred to the 5 mm NMR tube and stored at 4 ℃ for analysis. ^1^H-NMR data were acquired from Bruker Avance II-600 MHz spectrometer (Germany) with the reported methods in literatures [28]. The ^1^H-NMR spectrum was individually adjusted for zero, baseline and symmetrical calibration and the signal ranged from 0 to 10 ppm (part per million) was recorded to export the original data.

### Statistics

The data that indicated in each figure legend were acquired from at least 3 independent experiments and presented as mean values + standard deviation (SD). Differences between groups were analyzed using a Student’s *t*-test. The differences were deemed statistically significant at *P* < 0.05.

### Electronic supplementary material

Below is the link to the electronic supplementary material.

Supplementary Material 1

Supplementary Material 2

Supplementary Material 3

Supplementary Material 4

Supplementary Material 5

Supplementary Material 6

Supplementary Material 7

Supplementary Material 8

Supplementary Material 9

Supplementary Material 10

Supplementary Material 11

Supplementary Material 12

Supplementary Material 13

## Data Availability

The RIC-seq datasets generated for this study can be found in the Gene Expression Omnibus under accession GSE221551. The authors declare that the main data supporting the findings of this study are available within the article and its Supplementary Information files.

## Abbreviations

CRC: Colorectal cancer
GO: Gene ontology
IgG: Immunoglobulin G
IHC: Immunohistochemistry
MP-RNA: Multiple partner RNA
NSP-RNAs: Non-overlapping single partners
PMSF: Phenylmethylsulfonyl fluoride
PNK: Polynucleotide kinase
RIC-seq: RNA in situ conformation sequencing
RIP: RNA immunoprecipitation
RISC: RNA-induced silencing complex
RRIs: RNA-RNA interactions
SP-RNA: Single partner RNA

## Declarations

### Novelty & Impact

This report contributes to the mapping of RNA-RNA interactions (RRIs) by a newly established RNA in situ conformation sequencing (RIC-seq) technology in adenoma-carcinoma transition of human colorectal cancer (CRC). A lot of unexpected but interesting phenomena about RRIs in CRC were firstly revealed, including multiple partners RNAs, single partner RNA, homophilic RNA interactions among same family genes. The focused investigation on one antisense RNA OIP5-AS1 further shows a novel metabolic reprogramming mechanism of pyruvate.
