# Supplementary material for: The selective sponging of miRNAs by OIP5-AS1 regulates metabolic reprogramming of pyruvate in adenoma-carcinoma transition of human colorectal cancer
Source: BMC Cancer. 2024 May 21;24:611. doi: 10.1186/s12885-024-12367-7 (PMC11106987; doi:10.1186/s12885-024-12367-7)

**Supplementary data file 7-ShRNAs and OIP5-AS1**

The sequence information about LDHA shRNA, GPT shRNA, PDHA2 shRNA and OIP5-AS1 knock out were listed. The OIP5-AS1 knockout cells were verified by PCR and Sanger sequencing.

**LDHA shRNA**

Target sequence (5’UTR): GCACATTCTGAATCTTCTAGC

Top Strand

5'- CACCGCACATTCTGAATCTTCTAGCCGAAGCTAGAAGATTCAGAATGTGC -3'

Bottom Strand

5'- AAAAGCACATTCTGAATCTTCTAGCTTCGGCTAGAAGATTCAGAATGTGC -3'

**GPT shRNA**

Target sequence (5’UTR): GGTAGGGCCTTTACCTTTAAC

Top Strand

5'- CACCGGTAGGGCCTTTACCTTTAACCGAAGTTAAAGGTAAAGGCCCTACC -3'

Bottom Strand

5'- AAAAGGTAGGGCCTTTACCTTTAACTTCGGTTAAAGGTAAAGGCCCTACC -3'

**PDHA2 shRNA**

Target sequence (5’UTR): GCATGCTGCTAATCTTCATCA

Top Strand

5'- CACCGCATGCTGCTAATCTTCATCACGAATGATGAAGATTAGCAGCATGC -3'

Bottom Strand

5'- AAAAGCATGCTGCTAATCTTCATCATTCGTGATGAAGATTAGCAGCATGC -3'

**OIP5-AS1 knock out**


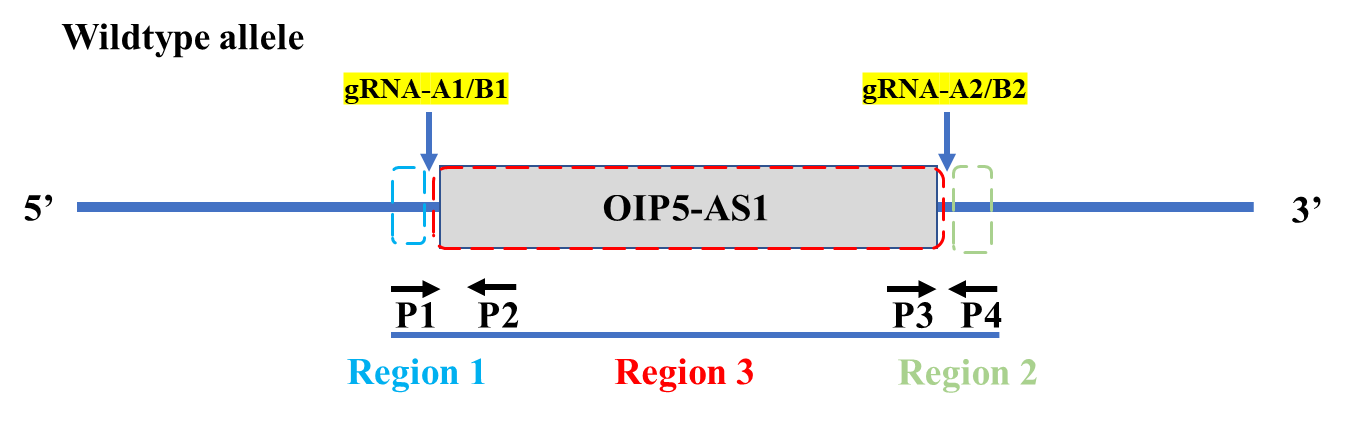


**Primer sequences of sgRNAs**

sgRNA-A1: CCTGGCGTGTATTCGAAAGGG

sgRNA-A2: CTGTTCAGGTAACGAGTGCGGG

sgRNA-B1: ACTCACTGGAGAAGACCACGGG

sgRNA-B2: CTTTCCCGAACGCTTCACTGGG

**To verify wild type (WT), we use primers P1+P2 for** **Region 1 or P3+P4 Region 2**

**Primers for Region 1**

Forward: ACCTTGAGAAGCTGCGAAGATGG

Reverse: TTCTGCTTGCAAAATGATGTCT

**Primers for Region 2**

Forward: TTATAGTGCTTAACCTGGAAACAA

Reverse: ATAAGCAGTCACACAGGCCATAGGAC

**To verify *OIP5-AS1* knockout (KO) phenotype, we use primers P1+P4 for Region 3**

**Primers for Region 3**

Forward: ACCTTGAGAAGCTGCGAAGATGG

Reverse: ATAAGCAGTCACACAGGCCATAGGAC

5’ arm (P1+P2), verification of OIP5-AS1 knockout cells.


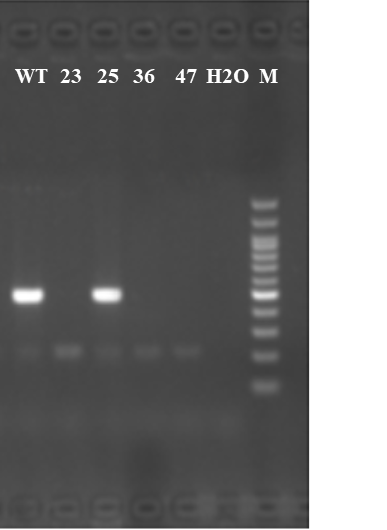

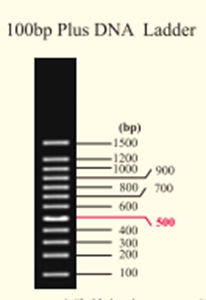


3’ arm (P3+P4), verification of OIP5-AS1 knockout cells.


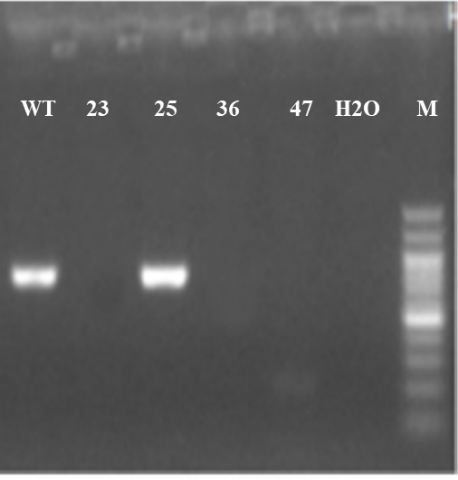

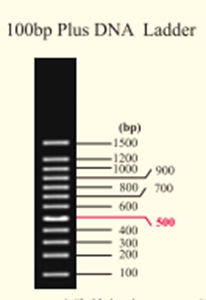


full length (P3+P4), verification of OIP5-AS1 knockout cells.

  **
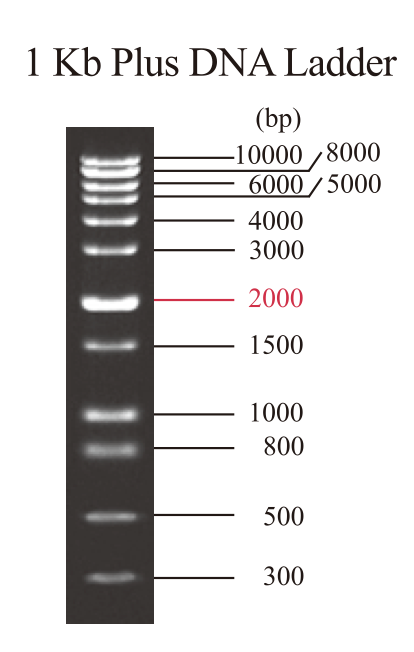
**

**Sanger sequencing of OIP5-AS1 knockout**

agggttggccaccttgagaagctgcgaagatgg-- del4968bp—ttatagtgcttaacctggaaacaaatgtactacagcccca


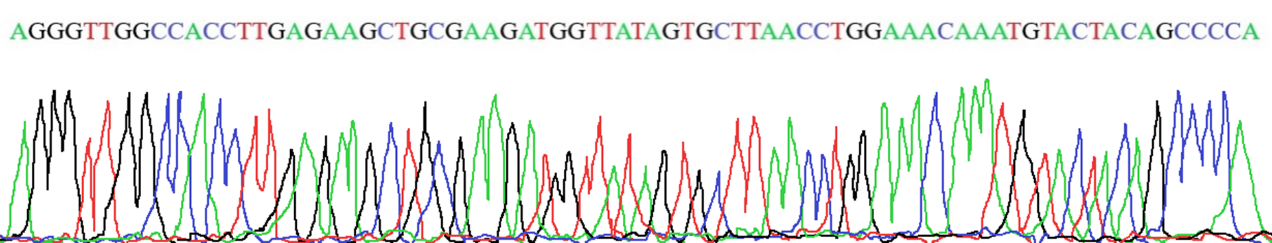

Supplement: Supplementary file 7 — Supplementary Material 7 [file 12885_2024_12367_MOESM7_ESM.docx]
