# Supplementary material for: The selective sponging of miRNAs by OIP5-AS1 regulates metabolic reprogramming of pyruvate in adenoma-carcinoma transition of human colorectal cancer
Source: BMC Cancer. 2024 May 21;24:611. doi: 10.1186/s12885-024-12367-7 (PMC11106987; doi:10.1186/s12885-024-12367-7)
Supplement: Supplementary file 8 — Supplementary Material 8 [file 12885_2024_12367_MOESM8_ESM.docx]

**
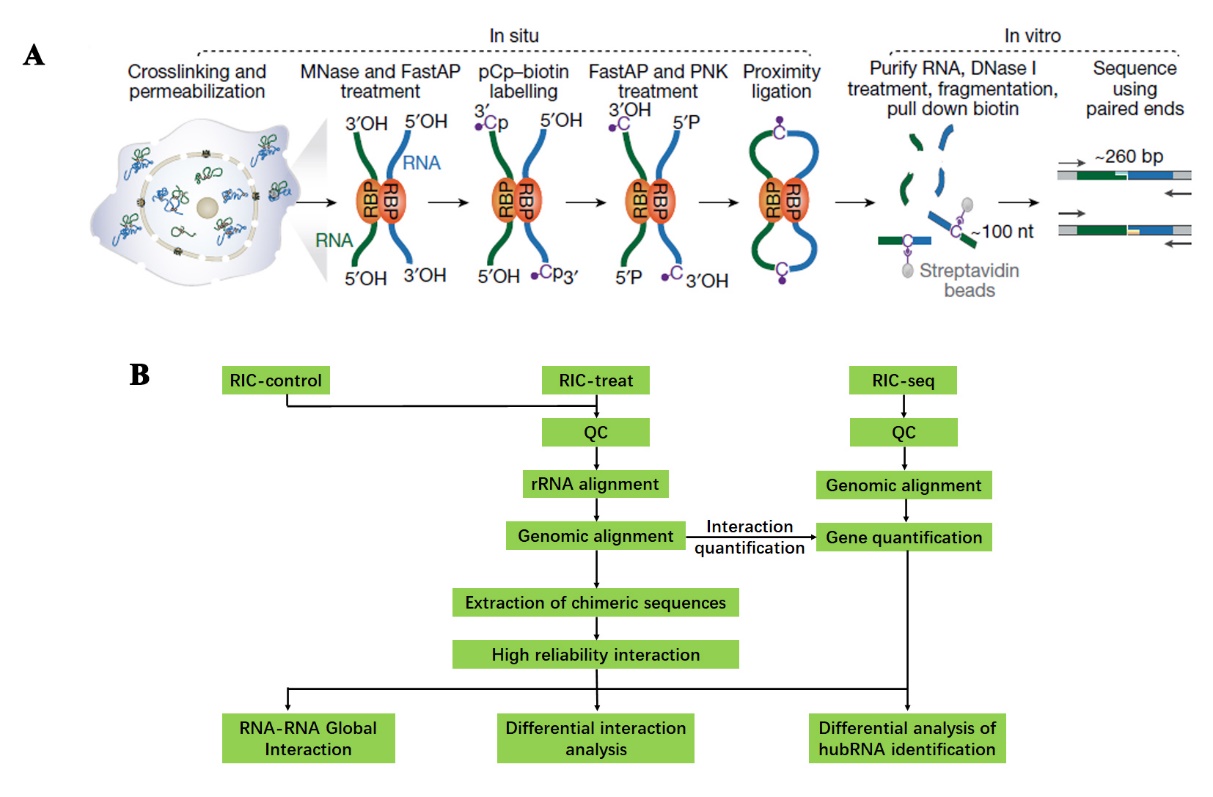
**

**Supplementary figure 1. Analysis of RNA-RNA interaction by RIC-seq.**

The recently developed RIC-seq capture high-level RNA structures and molecular interaction sites at the cellular in situ level. **A.** Principle of RIC-seq technology [9]. **B.** Data analysis flow of RIC-seq.


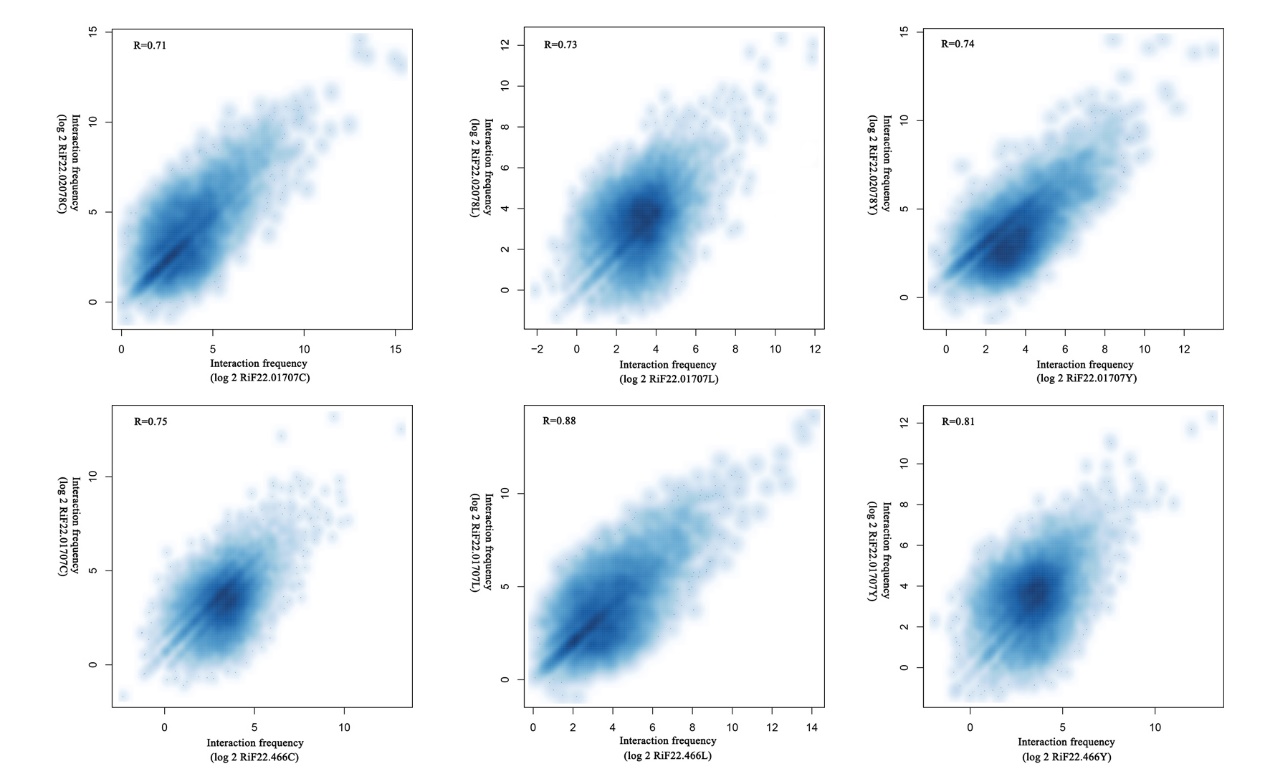


**Supplementary figure 2.** **Correlation analysis of repeated samples.**

The samples were divided into three groups colorectal adenoma (L), cancerous tissues (C), and tissues distal to cancer (Y), with two replicates for each group. The representative results of the correlation analysis of the two replicates were shown.


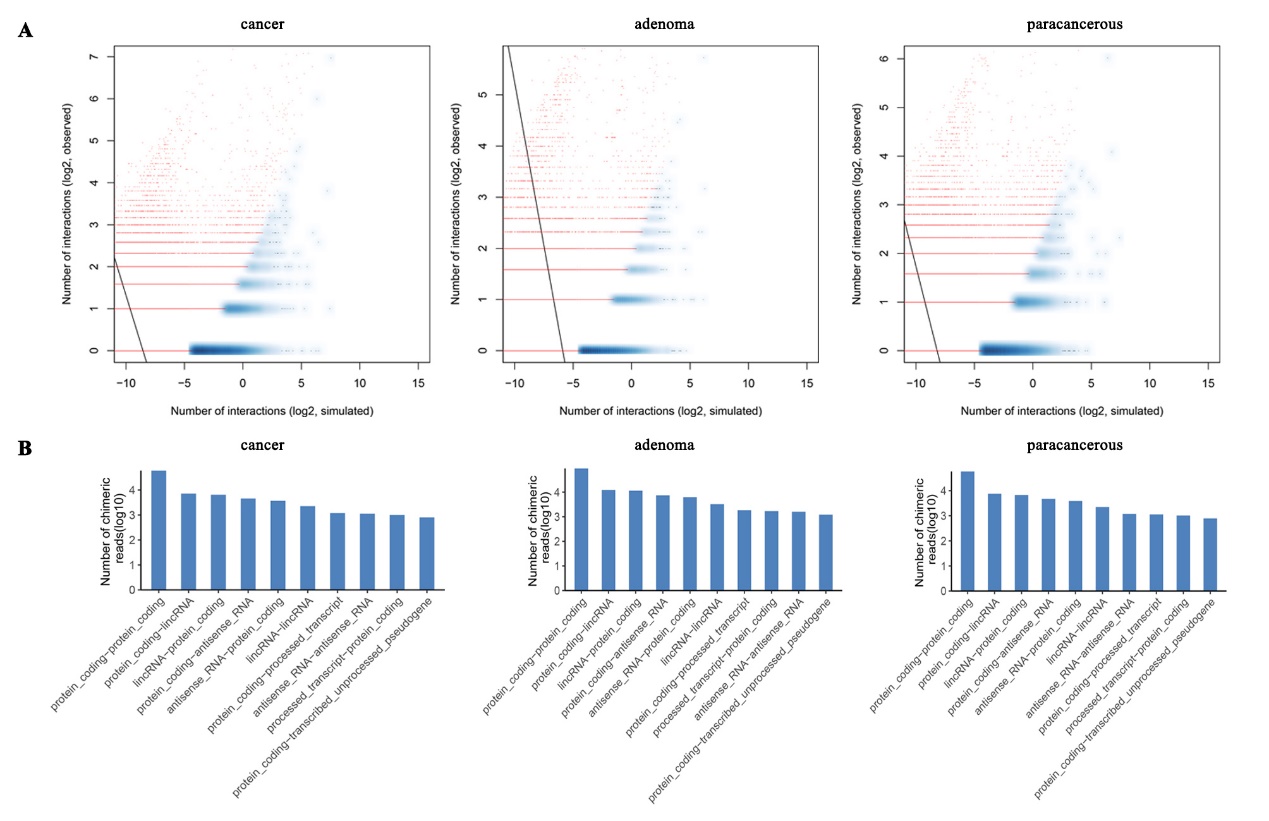


**Supplementary figure 3. Global view of RNA–RNA interactions.**

**A.** The RNA–RNA interactions in cancer (n=2,039,729), adenoma (n=1,871,901) and paracancerous tissues (n=1,937,482) were plotted against the average pairwise interaction counts from 100,000 simulations. **B.** RNA–RNA interaction types and their numbers of RIC-seq chimeric reads.


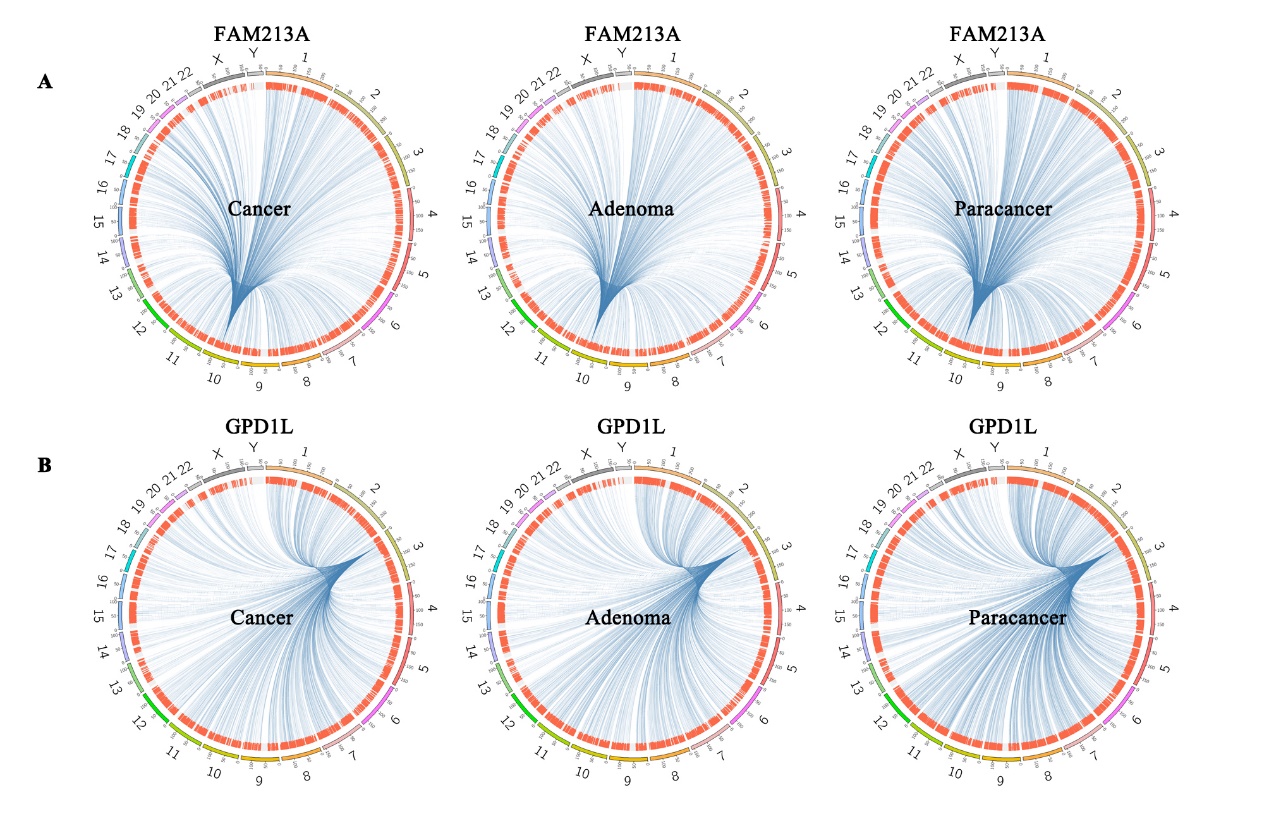


**Supplementary figure 4. Visualization of interaction for multi-CP RNA.**

The interacting RNAs of FAM213A **(A)** and GPD1L **(B)** were showed by circos plot. The outer circles show different chromosomes, and the inner circles show unique contacts.


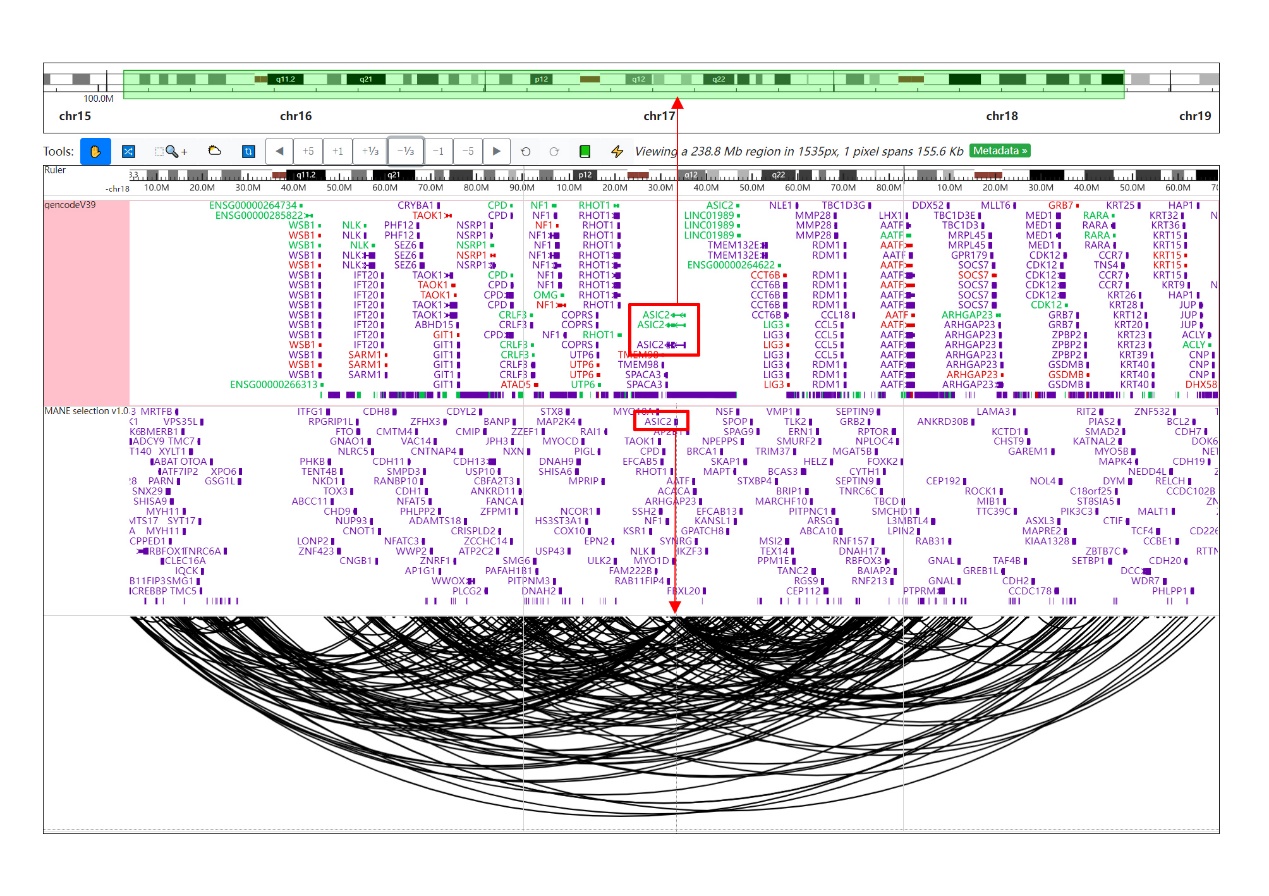


Supplementary figure 5 WashU track of RNA interactions.

The RIC-Seq data were imported into WashU (https://epigenomegateway.wustl.edu/browser/), and the track site was navigated near ASIC2 (gencodeV39 chr17:33013086-33293295) to visualize interactions. The arrows showed the ASIC2 position on chromosome 17 and the site of interactions.


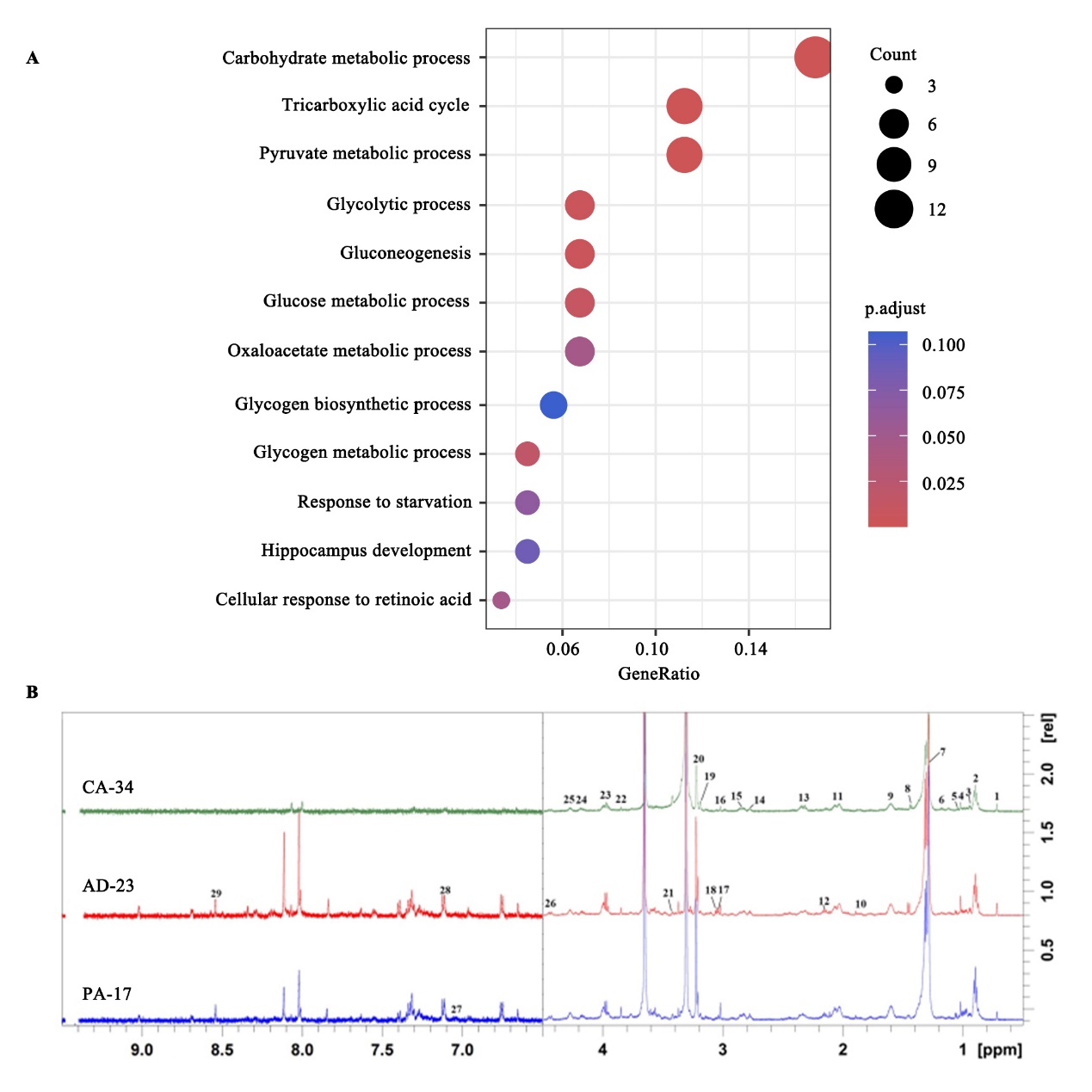


**Supplementary figure 6. GO enrichment and metabolites ^1^HNMR analysis**

**A.** The targets of miRNA-873, miRNA-335 and miRNA-323a and the interacting partners of targets in RIC-seq were analyzed by Gene Ontology enrichment. **B.** The metabolites of measured by ^1^HNMR analysis. Through literature review and comparison of standard product library, the metabolites were identified as: 1.Bile acids(0.69-0.74ppm); 2.Very Low Density Lipoprotein(0.82-0.928 ppm); 3. Leucine(0.928-0.95 ppm); 4.Isoleucine(0.95-1.01 ppm); 5.Valine(1.01-1.03 ppm); 6. Hydroxybutanoic acid(1.08-1.2 ppm); 7.Lactic acid(1.2-1.41 ppm); 8.Alanine(1.41-1.48 ppm); 9.Citrullin(1.55-1.67 ppm); 10.Acetic acid(1.88-1.91 ppm); 11.Proline(2-2.09 ppm); 12.Glutamic Acid(2.29-2.38 ppm); 13.pyruvate(2.38-2.52 ppm); 14.Dimethylamine(2.73-2.8 ppm); 15.Aspartic acid(2.8-2.88 ppm); 16.Creatinine(3-3.028 ppm); 17.Creatine(3.028-3.04 ppm); 18.Disodium2-oxoglutaratedihydrate(3.04-3.08 ppm); 19.Choline hydroxide(3.17-3.19 ppm); 20.Oxyneurine(3.2-3.24 ppm); 21.Taurine(3.39-3.45 ppm); 22.α-glucose(3.8-3.88 ppm); 23.Arginine(3.92-4.04 ppm); 24.Creatinine(4.1-4.23ppm); 25.Threonine(4.24-4.32ppm); 26.Choline glycerophosphate(4.42-4.46ppm); 27.Histidine(7.08-7.15ppm); 28.Phenylalanine(7.28-7.37 ppm); 29.Methanoic acid(8.52-8.56 ppm).


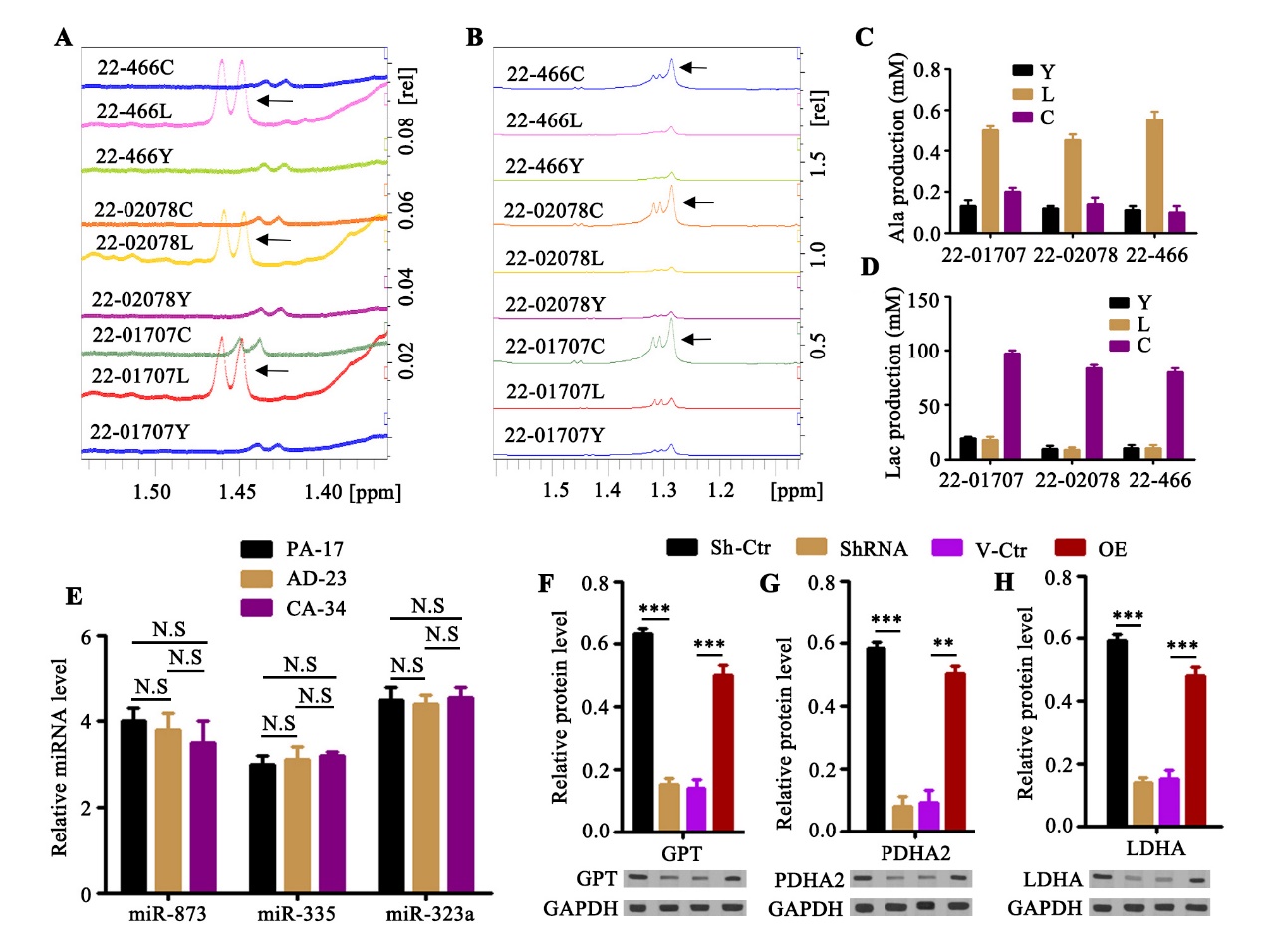


**Supplementary figure 7. Metabolites in clinical samples.**

**A and B**. The signal in relative chemical shifts between 1.40-1.50 ppm (**A**, alanine) and 1.20-1.40ppm (**B**, lactic acid) in colorectal cancer patients 22-01707, 22-02078 and 22-466 were selected to export the original data. The arrows show the sites of metabolites with density variation. **C and D**. The production of alanine (C, Ala) and lactic acid (D, Lac) in colorectal cancer patients 22-01707, 22-02078 and 22-466 were detected by Colorimetric Assay kits. Y, paracancer; L, adenoma; C, cancer. **E**. The RNA expression of miR-873, miR-335 or miR-323a in PA-17, AD-23 or CA-34 cells was evaluated by qRT-PCR. Data are means ± SD from three independent experiments. N.S, no significance. **F, G and H**. The GPT **(**F**)** shRNA (ShRNA), PDHA2 (G) shRNA, LDHA (H) shRNA or shRNA control (Sh-Ctr) plasmids were individually transfected into AD-23, PA-17 or CA-34 cells and selected by puromycin. The stable clone cells with GPT shRNA, PDHA2 shRNA or LDHA shRNA were transiently transfected with GPT, PDHA2 or LDHA cDNA (OE) or vector control (V-Ctr) plasmids. The relative protein levels were quantified and normalized to GAPDH. The original gel data were showed in supplementary figure 9. Data are means ± SD from three independent experiments. ***P < 0.001, **P < 0.01.


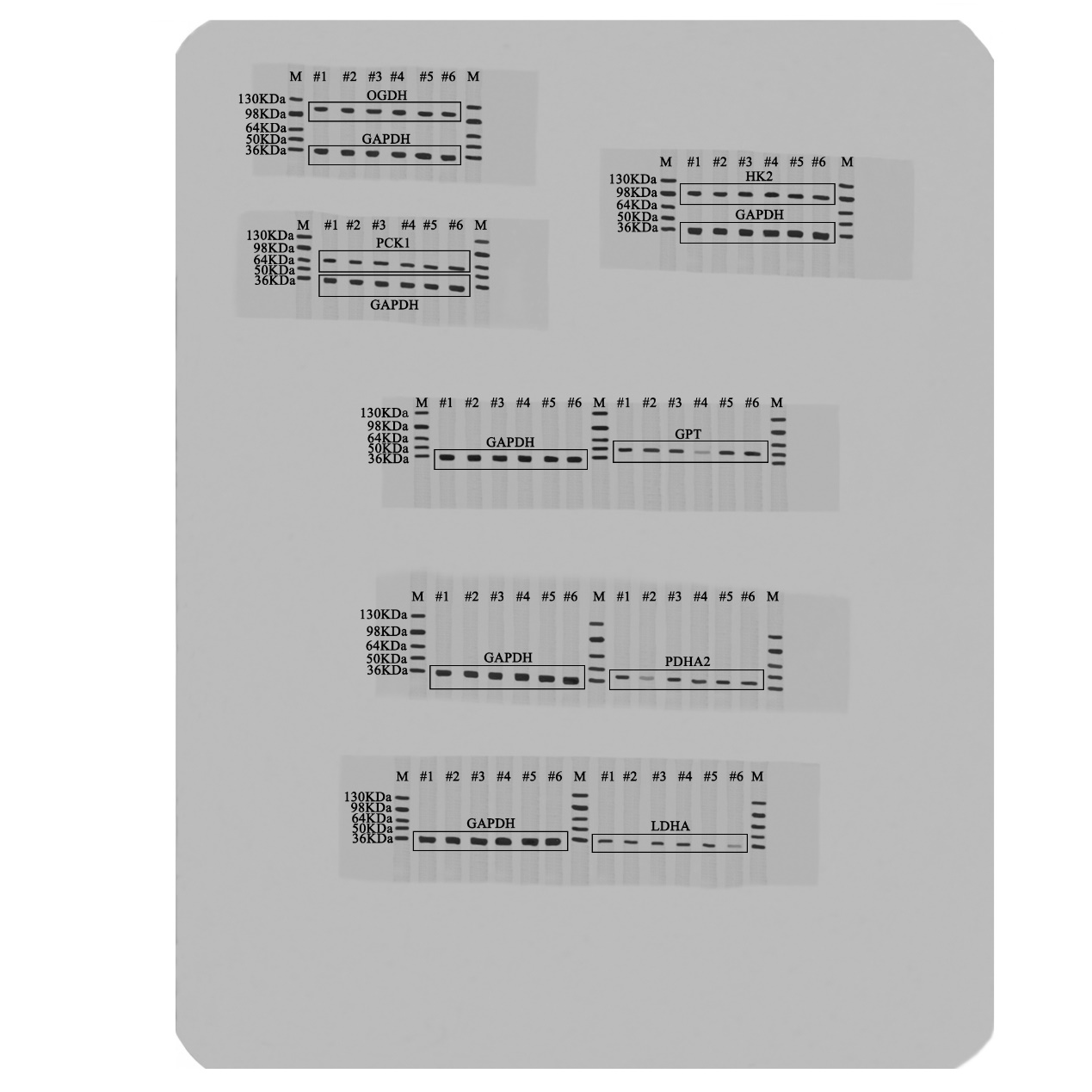


**Supplementary figure 8.**

The full images of western blotting data for Fig. 5A-5F. During the protein transferring, the nitrocellulose membranes were cut to fit with the SDS-PAGE gels. After incubation with secondary antibodies, the nitrocellulose membranes were placed onto one whole Eastman Kodak Co. hyper film for uniform exposure. The blots within the frame were cropped in main paper. Lane M, protein marker; Lane #1, OIP5-AS1 wild type PA-17 cells; Lane #2, OIP5-AS1 knocking out PA-17 cells; Lane #3, OIP5-AS1 wild type AD-23 cells; Lane #4, OIP5-AS1 knocking out AD-23 cells; Lane #5, OIP5-AS1 wild type CA-34 cells; Lane #6, OIP5-AS1 knocking out CA-34 cells.


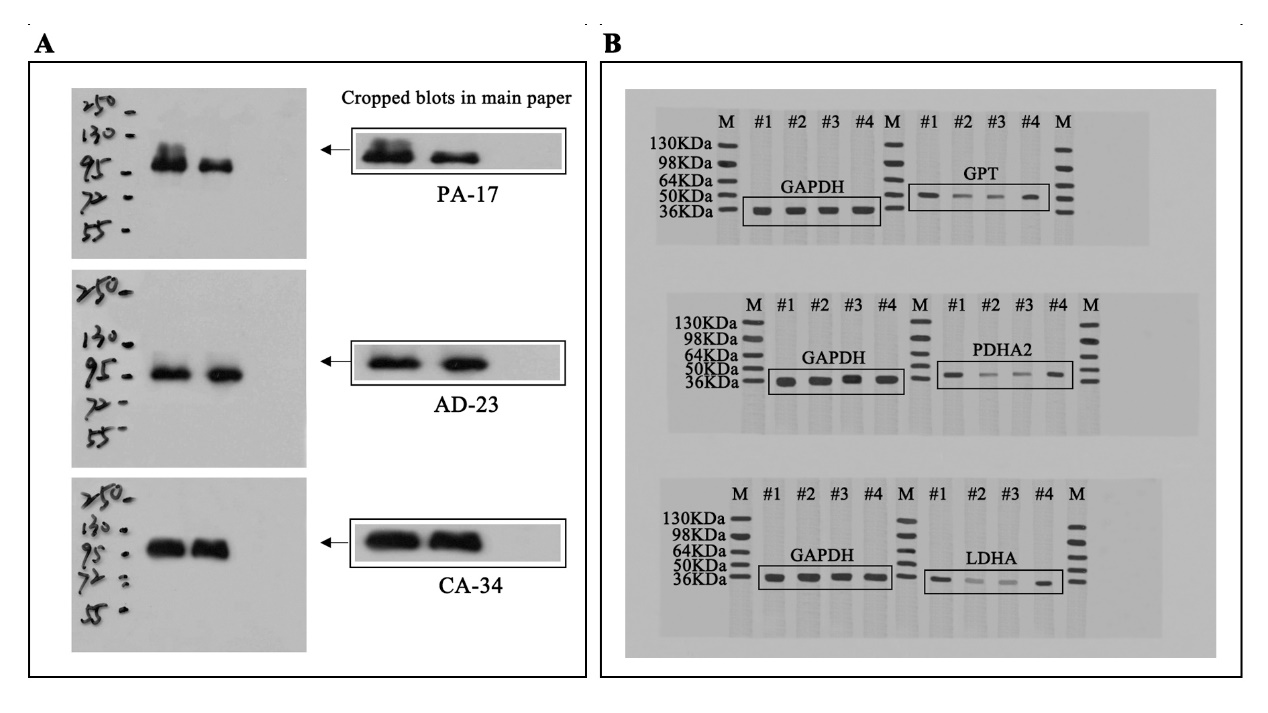


**Supplementary figure 9.**

**A.** The full images of western blotting data for Fig. 3F. **B.** The full images of western blotting data for Supplementary figure 7F-7H. During the protein transferring, the nitrocellulose membranes were cut to fit with the SDS-PAGE gels. After incubation with secondary antibodies, the nitrocellulose membranes were placed onto one whole Eastman Kodak Co. hyper film for uniform exposure. The blots within the frame were cropped in main paper. Lane M, protein marker; Lane #1, shRNA control; Lane #2, shRNA; Lane #3, vector control; Lane #4, cDNA transfected cells.
