# Supplementary material for: The selective sponging of miRNAs by OIP5-AS1 regulates metabolic reprogramming of pyruvate in adenoma-carcinoma transition of human colorectal cancer
Source: BMC Cancer. 2024 May 21;24:611. doi: 10.1186/s12885-024-12367-7 (PMC11106987; doi:10.1186/s12885-024-12367-7)
Supplement: Supplementary file 9 — Supplementary Material 9 [file 12885_2024_12367_MOESM9_ESM.pdf]

Supplementary table 1 General statistics by FastQC

Sample name	%Dups	%GC	M Seqs
RiF22-01707C	71.50%	53%	97.5
RiF22-01707L	72.90%	48%	93.5
RiF22-01707Y	86.40%	49%	98
RiF22-02078C	71.20%	41%	107.3
RiF22-02078L	83.40%	47%	99.3
RiF22-02078Y	78.80%	46%	86.1
RiF22-466C	73.40%	52%	94.6
RiF22-466L	78.20%	46%	96.1
RiF22-466Y	77.40%	43%	106.2

% Dups, Ratio of repeated reads; % GC, GC content;
M Seqs, Total sequencing
