# Supplementary material for: The selective sponging of miRNAs by OIP5-AS1 regulates metabolic reprogramming of pyruvate in adenoma-carcinoma transition of human colorectal cancer
Source: BMC Cancer. 2024 May 21;24:611. doi: 10.1186/s12885-024-12367-7 (PMC11106987; doi:10.1186/s12885-024-12367-7)
Supplement: Supplementary file 10 — Supplementary Material 10 [file 12885_2024_12367_MOESM10_ESM.pdf]

Supplementary table 2 All map reads by STAR program

Sample	IR	RL	UMRs %	RUs: too short%	RUs: other%	% of chimeric reads
RiF22-01707L	86801750	145	91.23%	3.95%	0.32%	0.54%
RiF22-01707Y	43016370	139	61.06%	33.32%	0.27%	0.97%
RiF22-01707C	44134564	140	62.06%	32.46%	0.29%	0.86%
RiF22-02078L	87759209	146	91.60%	3.67%	0.34%	0.59%
RiF22-02078Y	51343815	142	77.55%	16.81%	0.35%	0.90%
RiF22-02078C	42487988	138	74.93%	19.27%	0.32%	0.92%
RiF22-466L	43991109	140	78.24%	16.05%	0.34%	0.66%
RiF22-466Y	49923531	140	75.87%	18.34%	0.33%	0.93%
RiF22-466C	43221589	138	67.90%	26.63%	0.27%	1.08%

IR,Input reads; RL,Average input read length; UMRs,Uniquely mapped reads;RU,reads unmapped
