# Supplementary material for: The selective sponging of miRNAs by OIP5-AS1 regulates metabolic reprogramming of pyruvate in adenoma-carcinoma transition of human colorectal cancer
Source: BMC Cancer. 2024 May 21;24:611. doi: 10.1186/s12885-024-12367-7 (PMC11106987; doi:10.1186/s12885-024-12367-7)
Supplement: Supplementary file 11 — Supplementary Material 11 [file 12885_2024_12367_MOESM11_ESM.pdf]

Supplementary table 3 RNA types involved in RNA interactions

RNA type	cancer	adenoma	paracancer
unprocessed_pseudogene	78	70	88
unitary_pseudogene	1	2	26
transcribed_unprocessed_pseudogen	152	179	148
transcribed_processed_pseudogene	0	0	50
TR_J_gene	1	0	0
TR_V_gene	0	0	1
TEC	6	21	7
snRNA	2	1	
snoRNA	1	2	1
sense_overlapping	36	32	45
sense_intronic	15	25	29
ribozyme	1	1	0
protein_coding	6986	6141	8285
processed_transcript	178	177	212
processed_pseudogene	44	36	44
polymorphic_pseudogene	3	1	2
misc_RNA	3	4	5
miRNA	1	1	1
macro_lncRNA	1	1	1
lincRNA	762	729	944
IG_V_pseudogene	0	0	1
IG_V_gene	3	2	1
bidirectional_promoter_lncRNA	3	1	2
antisense_RNA	476	488	516
3prime_overlapping_ncRNA	2	4	1
total	8755	7918	10410
