# Supplementary material for: The selective sponging of miRNAs by OIP5-AS1 regulates metabolic reprogramming of pyruvate in adenoma-carcinoma transition of human colorectal cancer
Source: BMC Cancer. 2024 May 21;24:611. doi: 10.1186/s12885-024-12367-7 (PMC11106987; doi:10.1186/s12885-024-12367-7)
Supplement: Supplementary file 12 — Supplementary Material 12 [file 12885_2024_12367_MOESM12_ESM.pdf]

Supplementary table 4 multi-CP RNAs in different tissues

RNA type	RNA name	C-CPs	A-CPs	P-CPs
protein_coding	ASIC2	552	760	402
protein_coding	GPD1L	416	489	340
protein_coding	FAM213A	379	415	185
protein_coding	SLC16A14	219	156	89
protein_coding	TRAM1	213	90	91
protein_coding	MBP	151	156	124
protein_coding	WDR62	115	59	59
protein_coding	NCKAP5	102	137	101
protein_coding	TGFBR3	100	36	49
protein_coding	ARID4B	89	118	62
misc_RNA	RN7SL5P	78	31	37
protein_coding	CPT1A	68	90	34
protein_coding	DLG2	62	99	69
ribozyme/lincRNA	RMRP	58	32	24
protein_coding	PHKB	58	73	34
processed_transcript	MIR663AHG	54	11	21
lincRNA	AJ009632.2	52	21	19
protein_coding	POLA2	51	40	18
protein_coding	PCDH11X	48	47	17
protein_coding	CDKL1	43	23	29
protein_coding	TASP1	41	46	10
protein_coding	OPRD1	40	7	11
protein_coding	PLOD1	32	3	12
protein_coding	PTPRD	30	23	15
unprocessed_pseudogene	TSPY20P	25	24	13
processed_pseudogene	AL117692.1	24	12	13
lincRNA	MIR3681HG	24	30	27
protein_coding	RBFOX1	7	19	22
protein_coding	PARP16	23	21	17
protein_coding	DPP10	23	18	20
protein_coding	ATP13A2	23	9	8
protein_coding	EPB41	21	6	12
protein_coding	SLCO1C1	20	4	26

C-CPs, couple numbers in cancerous tissue;A-CPs, couple numbers in adenomea tissue; P-CPs, couple numbers in paracancerous tissue
