# Supplementary material for: The selective sponging of miRNAs by OIP5-AS1 regulates metabolic reprogramming of pyruvate in adenoma-carcinoma transition of human colorectal cancer
Source: BMC Cancer. 2024 May 21;24:611. doi: 10.1186/s12885-024-12367-7 (PMC11106987; doi:10.1186/s12885-024-12367-7)
Supplement: Supplementary file 13 — Supplementary Material 13 [file 12885_2024_12367_MOESM13_ESM.pdf]

Supplementary table 5 miRNA targets in RIC-seq

miR-873 targets	miR-335 targets	miR-323a targets
ACLY	SLC25A44	HK2
DALT	OIP5-AS1	LDHA
OGDH	GPT	SLC22A23
IDH3G	ALDOA	OIP5-AS1
PDHA2	PGM3	SLC25A12
PDPR	RPE	EIF1
PCK1		ARL1
GYS1		TMEF2
GSK3B		NFYB
PHKG2		
SLC25A11		
OIP5-AS1		
